# Specific NuRD components are required for fin regeneration in zebrafish

**DOI:** 10.1186/1741-7007-12-30

**Published:** 2014-04-29

**Authors:** Catherine Pfefferli, Fritz Müller, Anna Jaźwińska, Chantal Wicky

**Affiliations:** 1 Department of Biology, University of Fribourg, Ch. du Musée 10, CH-1700 Fribourg, Switzerland

**Keywords:** NuRD, Blastema, Fin, Regeneration, Zebrafish

## Abstract

**Background:**

Epimorphic regeneration of a missing appendage in fish and urodele amphibians involves the creation of a blastema, a heterogeneous pool of progenitor cells underneath the wound epidermis. Current evidence indicates that the blastema arises by dedifferentiation of stump tissues in the vicinity of the amputation. In response to tissue loss, silenced developmental programs are reactivated to form a near-perfect copy of the missing body part. However, the importance of chromatin regulation during epimorphic regeneration remains poorly understood.

**Results:**

We found that specific components of the Nucleosome Remodeling and Deacetylase complex (NuRD) are required for fin regeneration in zebrafish. Transcripts of the chromatin remodeler *chd4a*/Mi-2, the histone deacetylase *hdac1*/HDAC1/2, the retinoblastoma-binding protein *rbb4*/RBBP4/7, and the metastasis-associated antigen *mta2*/MTA were specifically co-induced in the blastema during adult and embryonic fin regeneration, and these transcripts displayed a similar spatial and temporal expression patterns. In addition, chemical inhibition of Hdac1 and morpholino-mediated knockdown of *chd4a*, *mta2*, and *rbb4* impaired regenerative outgrowth, resulting in reduction in blastema cell proliferation and in differentiation defects.

**Conclusion:**

Altogether, our data suggest that specialized NuRD components are induced in the blastema during fin regeneration and are involved in blastema cell proliferation and redifferentiation of osteoblast precursor cells. These results provide *in vivo* evidence for the involvement of key epigenetic factors in the cellular reprogramming processes occurring during epimorphic regeneration in zebrafish.

## Background

In contrast to mammals, some vertebrates such as urodeles and teleost fish benefit from exceptional regeneration mechanisms. Zebrafish are able to regenerate different organs after injury, including heart, fins, retina, liver, and spinal cord, and have become a powerful model organism for regenerative studies [1-5]. The caudal fin displays rapid and robust regeneration, and therefore provides a well-established system to study appendage regeneration in vertebrates [4,6,7].

The caudal fin of zebrafish is constituted of 16 to 18 bony fin rays (lepidotrichia), covered by an epidermis, and interconnected by soft inter-ray mesenchymal tissue [6]. Each individual bony ray consists of two concave hemirays that enclose a mesenchymal compartment composed of blood vessels, nerves, pigment cells, fibroblasts, and osteoblasts.

Upon amputation, the caudal fin is fully restored after approximately 3 weeks. This type of regeneration, called epimorphic regeneration, involves the formation of a blastema, a population of proliferating progenitor cells that arise from dedifferentiation of mesenchymal cells in the stump [4,8]. Regeneration of the caudal fin proceeds through three main steps: 1) wound healing, 2) blastema formation, and 3) regenerative outgrowth, including differentiation and patterning. Upon fin amputation, epidermal cells rapidly migrate to protect the wound and form a wound epidermis. Mesenchymal tissues in the stump then become disorganized, and cells start to proliferate and migrate distally, forming a blastema after approximately 24 to 48 hours post-amputation (hpa). During regenerative outgrowth, the blastema progenitor cells are maintained at the distal margin, while their daughter cells progressively redifferentiate in the proximal part of the fin regenerate. During this later phase, the fin regenerate can be subdivided into several compartments with distinct cellular and molecular properties [9-11].

The exact origin of blastema cells still remains unresolved. Recently, genetic cell-fate tracing studies have shown that the blastema is composed of a heterogeneous population of cells with restricted lineage fate and different tissue origin [12-14]. Thus, regeneration is achieved without cellular transdifferentiation. However, genetic ablation studies of osteoblasts prior to amputation have revealed that new bones are able to regenerate from non-osteoblast cells, suggesting that other cell types are plastic, and can transdifferentiate into osteoblasts to promote bone regeneration [15].

Animals with robust regenerative capacities are characterized by their flexibility to change gene expression in response to amputation. This cellular plasticity allows temporal suppression of differentiation genes and reactivation of developmental signaling pathways, which are required for the reconstitution of lost tissues [11,16-28].

Regulation of the chromatin structure is an important epigenetic mechanism, which has a direct influence on many biological processes. The Nucleosome Remodeling and Deacetylase (NuRD) complex is a multi-subunit complex widely expressed and evolutionarily conserved in animals and plants [29]. This complex is able to couple two important enzymatic functions: an ATP-dependent nucleosome remodeling activity catalyzed by the chromodomain helicase DNA binding proteins CHD3/4, also called Mi-2α/β, and a deacetylase activity executed by the histone deacetylases HDAC1/2 [30-33]. Additionally, the NuRD complex is also constituted of other non-catalytic subunits, including the methyl-CpG-binding domain proteins MBD2/3, the retinoblastoma-binding proteins RBBP7/4, and the metastasis-associated proteins MTA1/2/3 [34]. The composition of the NuRD complex can also be changed by the incorporation of unique subunits, raising the possibility of functional specialization for these distinct complexes [35].

The NuRD complex has been shown to play important developmental roles in cell fate determination [34]. In *Caenorhabditis elegans*, the Mi-2 homolog LET-418 is required for proper differentiation of the vulva [36] and for repression of germline-specific genes in somatic cells [37]. In *Drosophila melanogaster*, dMi-2 is essential for embryogenesis and germ cell development [38]. Yoshida *et al*. [39] have demonstrated that in mammals, Mi-2β functions in self-renewal and lineage choice of hematopoietic stem cells [39]. In addition, embryonic stem cells deficient in *mbd3* can initiate differentiation, but are not able to commit to specific lineages [40].

In this study, we investigated the potential role of the Mi-2/NuRD complex during fin regeneration in zebrafish. The zebrafish genome encodes several orthologs for every member of the vertebrate NuRD complex. However, we found that only one of each is expressed during fin regeneration. The orthologs of the NuRD components *chd4a*/Mi-2, *hdac1*/HDAC1/2, *rbb4*/RBBP4/7, and *mta2*/MTA are all induced in the distal blastema during regeneration of the adult and embryonic caudal fin, and display similar expression patterns. Additionally, inhibition of these genes impairs regenerative outgrowth. Our data suggest that putative NuRD components are induced in the blastema during fin regeneration, and are involved in the maintenance of blastema cell proliferation and in redifferentiation during the regenerative outgrowth phase.

## Results

### One of the three Mi-2 orthologs, *chd4a*, is specifically expressed in the blastema during fin regeneration

Mi-2, which is the core ATPase of the NuRD complex, is essential for regeneration and neoblast differentiation in the planarian *Schmidtea mediterranea*[41]. We therefore investigated whether Mi-2 could also be involved in zebrafish fin regeneration. A BLAST search of the zebrafish genome database (National Center for Biotechnology Information) identified three genes, *chd4a* (Gene ID: 558344), *chd4b* (Gene ID: 560622), and *chd3* (Gene ID: 568230), which encode polypeptides with high similarity to human Mi-2 proteins, also called CHD4 (or Mi-2β) and CHD3 (or Mi-2α). Sequence alignment revealed high similarity between the three zebrafish Mi-2 homologs, with the main functional domains being conserved (see Additional file 1: Figure S1). Chd4a and Chd4b share 82% identity, while Chd3 shares 66% identity with Chd4a and Chd4b. Moreover, Chd4a contains an additional domain, the AP endonuclease family 2 domain (AP2Ec) (see Additional file 1: Figure S1), which is not present in other Mi-2 orthologs. This evolutionarily conserved domain is associated with DNA damage repair and maintenance of genome stability [42].

**Figure 1:**
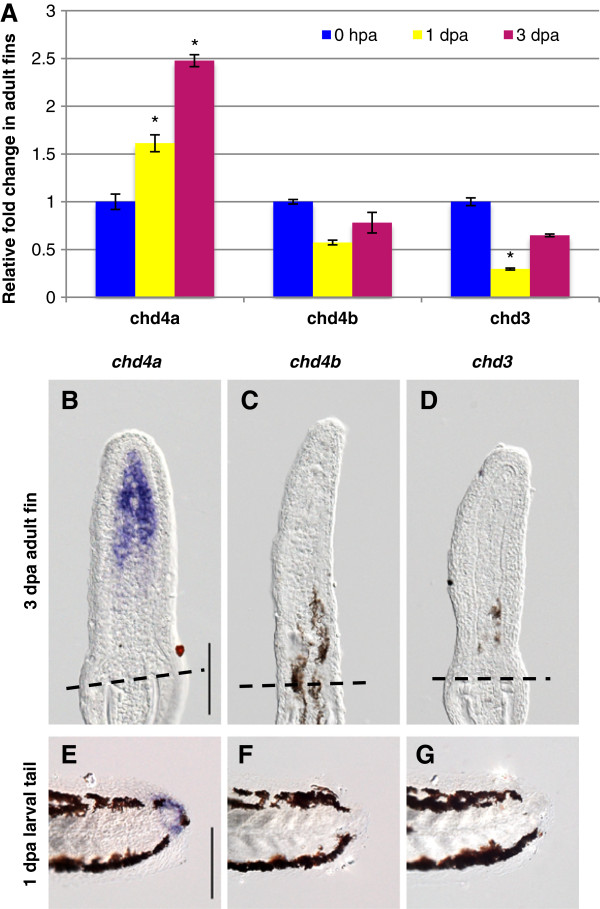
**One of the three Mi-2 orthologs, *chd4a*, is specifically induced in the blastema during fin regeneration. (A)** Quantitative real-time-PCR analysis of *chd4a*, *chd4b*, and *chd3* mRNA in regenerating adult caudal fins at 1 day post-amputation (dpa) and 3 dpa relative to control fins at 0 hours post-amputation (hpa). Error bars represent the SEM. *P < 0.001 **(B-D) ***In situ* hybridization with *chd4a ***(B)**, *chd4b ***(C)**, and *chd3 ***(D)** mRNA antisense probes on cryosections of regenerating adult caudal fins at 3 dpa. Dashed lines indicate the amputation plane. **(E-G)** Whole-mount *in situ* hybridization with *chd4a ***(E)**, *chd4b***(F)**, and *chd3***(G)** antisense probes in 1 dpa larval tails. Scale bar: 100 μm.

To determine whether these putative Mi-2 orthologs might play a role in fin regeneration, we first examined their expression profiles during this process. Quantitative real-time-PCR (qRT-PCR) identified significant upregulation of *chd4a* transcripts in regenerating fins at 3 days post-amputation (dpa) compared with amputated fins collected immediately after amputation (0 hpa) (Figure 1A). No upregulation was observed for the two other Mi-2 homologs, *chd4b* and *chd3,* in regenerating fins (Figure 1A). The temporal and spatial expression pattern of these genes was also analyzed by *in situ* hybridization (ISH) in regenerating adult caudal fins at 3 dpa. Consistent with the qRT-PCR data, only *chd4a* was induced in adult regenerating fins (Figure 1B; see Additional file 1: Figure S2). *chd4a* transcripts were specifically located in the blastema, but not in the adjacent epidermis (Figure 1B). No *chd4a* signal was detected in uninjured fins or during early stages of regeneration (8 and 24 hpa) (see Additional file 1: Figure S3). *chd4a* expression was initially weak, starting at 2 dpa during blastema formation (see Additional file 1: Figure S3). A robust signal was observed at 3 dpa, and then the expression persisted in the blastema of regenerating fins during regenerative outgrowth. By contrast, no signals were detected for the two other Mi-2 orthologs, *chd4b* and *chd3*, in the regenerating tissue (Figure 1C,D). In embryos, all three Mi-2 orthologs were expressed with slightly different expression patterns, suggesting that they have different functions during development (see Additional file 1: Figure S4).

Early zebrafish larvae are also able to regenerate their caudal fin folds after amputation with a similar mechanism to that of regenerating adult caudal fins [43,44]. Interestingly, *chd4a*, but neither *chd4b* nor *chd3*, was expressed in the mesenchymal cells of regenerating larval fin folds at 1 dpa (Figure 1E-G). Expression of *chd4a* mRNA is specific for regenerating fins, as it was not detected in uncut fin folds at the same developmental stage (3 days post-fertilization) (data not shown). Altogether, these results show that one of the three Mi-2 orthologs, *chd4a*, is transcriptionally induced in the blastema of regenerating adult and embryonic fins.

### Specific NuRD component orthologs are expressed in the blastema of regenerating fins

**Figure 2:**
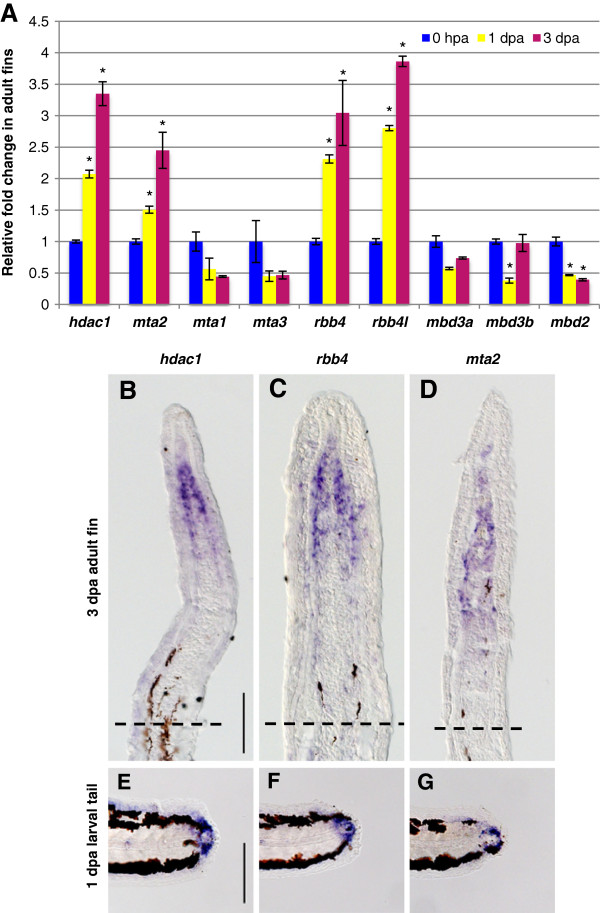
**Specific NuRD component orthologs are expressed in the blastema of regenerating fins. (A)** Quantitative real-time-PCR analyses of *hdac1*, *mta1*, *mta2*, *mta3*, *rbb4*, *rbb4l*, *mbd3a*, *mbd3b*, and *mbd2* mRNA in regenerating adult caudal fins at 1 and 3 dpa relative to control fins at 0 hpa. Error bars represent the SEM. *P < 0.001 **(B-D) ***In situ* hybridization with *hdac1 ***(B)**, *rbb4***(C)**, and *mta2 ***(D)**, antisense probes on cryosections of regenerating adult caudal fins at 3 dpa. Dashed lines indicate the amputation plane. **(E-G)** Whole-mount *in situ* hybridization with *hdac1 ***(E)**, *rbb4 ***(F)** and *mta2 ***(G)** antisense probes in 1 dpa larval tails. Scale bar: 100 μm.

We then investigated whether other NuRD components are also expressed during fin regeneration. The genome of zebrafish encodes orthologs for all components of the vertebrate NuRD complex. BLAST searches identified three MTA orthologs, LOC794477 (*mta1*) (Gene ID: 794477), *mta2* (Gene ID: 326078), and *mta3* (Gene ID: 100003254); two RBBP4/7 orthologs, *rbb4* (Gene ID: 321726) and *rbb4l* (Gene ID: 322129); one MBD2 ortholog, *mbd2* (Gene ID: 337105); and two MBD3 orthologs, *mbd3a* (Gene ID: 337133) and *mbd3b* (Gene ID: 321217); but only one HDAC1/2 ortholog, *hdac1* (Gene ID: 192302). We examined the expression profile of these genes to test whether NuRD components other than *chd4a* were also specifically expressed in adult regenerating fins. qRT-PCR analysis revealed that transcripts of *hdac1*, the two RBBP4/7 orthologs *rbb4* and *rbb4l*, and one of the three MTA orthologs, *mta2*, were significantly upregulated in adult regenerating fins at 3 dpa compared with 0 hpa, whereas no upregulation was observed for the other orthologs (Figure 2A).

qRT-PCR data were confirmed by ISH on cryosections of adult caudal fins at 3 dpa. A single RNA antisense probe was designed for the two RBBP4/7 orthologs *rbb4* and *rbb4l* because of their high RNA (75%) and amino acid (94%) sequence similarity. Positive signals for *hdac1*, *rbb4*, and *mta2* transcripts were detected in the blastema of adult regenerating fins, with an expression pattern similar to that of *chd4a* (Figure 2B-D). No signals were detected for the orthologs whose expression was not upregulated by qRT-PCR (data not shown). Furthermore, *hdac1*, *rbb4*, and *mta2* transcripts were also expressed in mesenchymal cells of regenerating larval fin folds at 1 dpa (Figure 2E-G). Thus, the overlapping expression pattern of some NuRD orthologs in fin regenerates raises the possibility that the expression of a specialized NuRD complex composed of Chd4a, Rbb4/Rbb4l, Hdac1, and Mta2 is specifically induced in the blastema during fin regeneration.

### Morpholino-mediated knockdown of *chd4a*, *mta2*, and the two RBBP4 orthologs *rbb4* and *rbb4l* impairs fin regeneration

**Figure 3:**
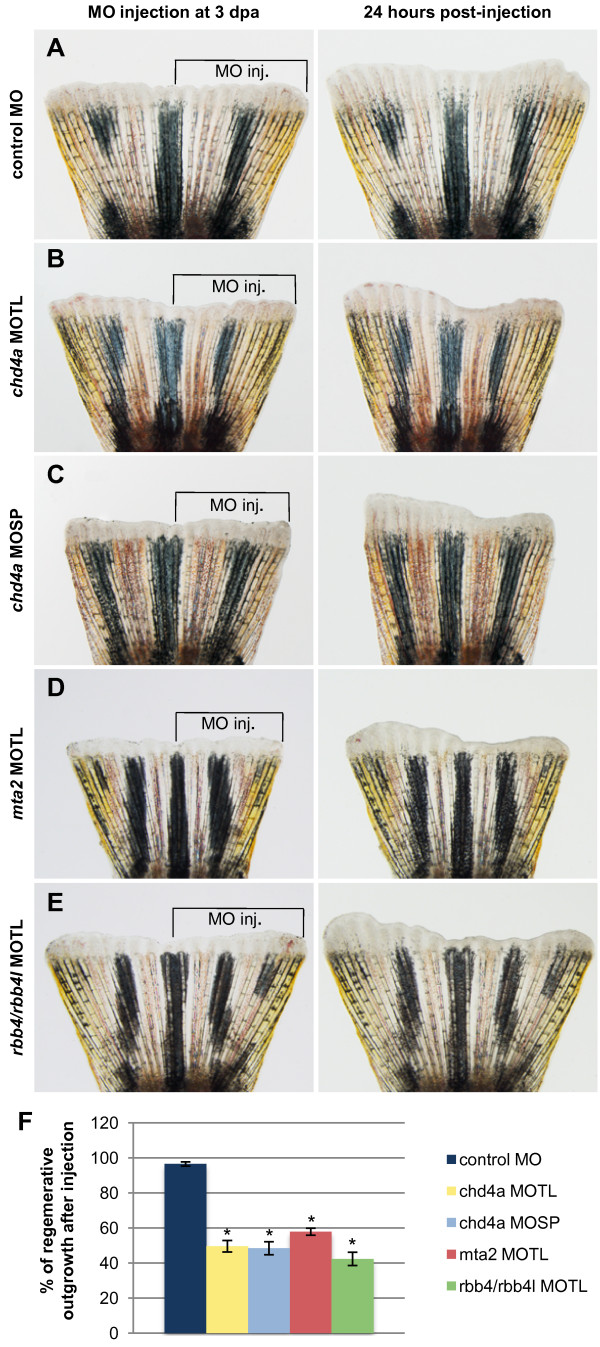
**Morpholino-mediated knockdown of *chd4a*, *mta2, *and the two RBBP4 orthologs *rbb4 *and *rbb4l *results in the reduction in regenerative outgrowth. (A-E)** The dorsal half of fins was injected with control **(A)**, *chd4a* translational blocking **(B)**, *chd4a* splice blocking **(C)**, *mta2* translational blocking **(D)**, and *rbb4* + *rbb4l* translational blocking **(E)** vivo-morpholinos (MOs). The uninjected ventral half was used as an internal control to assess normal outgrowth. The left panels show fins shortly after injection at 3 dpa and the right panels show the same fins at 24 hours post-injection (hpi). The control MO does not affect regenerative outgrowth compared with the uninjected ventral side of the fins. Injection of the antisense MOs specific for *chd4a*, *mta2*, or the two RBBP4 orthologs *rbb4* and *rbb4l* resulted in reduction in regenerative outgrowth compared with the uninjected side. **(F)** Percentage of regenerative outgrowth after MO injection relative to the uninjected control side of the fins. Error bars represent the SEM. n = 10. *P < 0.01.

To determine whether these putative NuRD components play a role in fin regeneration, expression of *chd4a*, *mta2*, and the two RBBP4 orthologs *rbb4* and *rbb4l* was knocked down using vivo-morpholinos (MOs). For *chd4a*, two different sets of antisense vivo-morpholinos were designed: a translational blocking MO (MOTL) and a splice blocking MO (MOSP). The efficacy of the splice blocking *chd4a* MOSP was tested in zebrafish embryos, and was found to specifically impair the splicing of *chd4a* transcript (see Additional file 1: Figure S5). MOs were injected into the dorsal half of adult regenerating fins at 3 dpa, and the uninjected ventral half was used as an internal control. The effects of the MOs were analyzed at 24 hours post-injection (hpi) by comparing the regenerative surfaces of the injected and uninjected fin halves. No significant differences in regeneration were observed in fin regenerate areas injected with the control MO compared with the uninjected areas (Figure 3A,F). However, injection of the translational (MOTL) or the splice (MOSP) blocking *chd4a* MOs resulted in a significant reduction in regenerative outgrowth compared with the uninjected region or with fin halves injected with the control MO (Figure 3B,C,F). Interestingly, injection of MOs specific for the metastasis-associated gene *mta2* (Figure 3D) or for the two retinoblastoma-binding orthologs *rbb4* and *rbb4l* (Figure 3E) also significantly decreased regenerative outgrowth compared with the uninjected fin halves (Figure 3F). Thus, morpholino-mediated knockdown of the NuRD components *chd4a*, *mta2*, and the two *rbb4* orthologs resulted in a significant reduction in regenerative outgrowth in adult caudal fins, suggesting an important role for these epigenetic factors during fin regeneration.

### Specific HDAC1 inhibition affects regenerative outgrowth

**Figure 4:**
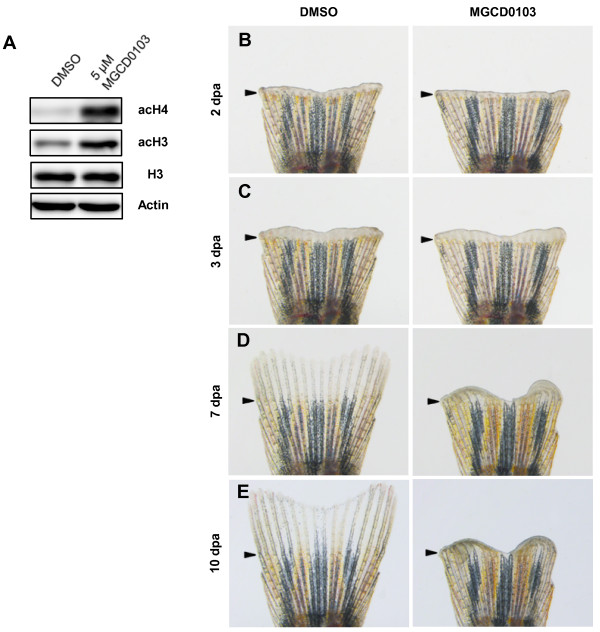
**Specific Hdac1 inhibition with MGCD0103 impairs fin regeneration. (A)** Western blot analysis for acetylation of histone H3 and histone H4 in 4 dpa fin regenerates treated with DMSO or 5 μM MGCD0103 starting from the time of amputation. The level of acetyl-H3 and acetyl-H4 in MGCD0103-treated fins was increased compared with DMSO-treated fins. β-actin and histone H3 were used as loading controls. **(B-E)** Whole caudal fins at 2 **(B)**, 3 **(C)**, 7 **(D)** and 10 dpa **(E)** treated with DMSO or 5 μM MGCD0103 starting from the time of amputation. MGCD0103 treatment for 10 days impaired regenerative growth without affecting the early stages of regeneration. Arrowheads indicate the amputation plane.

To investigate the function of the histone deacetylase Hdac1 during fin regeneration, we used a pharmacological approach to target its activity. Hdac1 is the only HDAC1/2 ortholog encoded by the genome of zebrafish, and is required for development of the retina, the neural crest, and the central nervous system [45-50]. In humans, HDAC1 and HDAC2 can be selectively inactivated with MGCD0103 (Mocetinostat), a class I-specific HDAC inhibitor [51,52]. Sequence alignment revealed that the catalytic domain of zebrafish Hdac1 is highly conserved (93% identity with the catalytic domain of human HDAC1 and HDAC2), suggesting that MGCD0103 might also be functional in zebrafish (see Additional file 1: Figure S6). In contrast to morpholinos, which have to be injected into the regenerating tissue, chemical inhibitors can be added directly into the fish water. To inhibit Hdac1 activity during fin regeneration, fish were treated with 5 μM MGCD0103 for 10 days after fin amputation, or with 0.05% DMSO as control. The specificity of MGCD0103 treatment was evaluated by measuring the global acetylation levels of histones H3 and H4 in fin regenerates by western blot analysis. We found that the levels of acetylated histones H3 and H4 were significantly increased in fin regenerates treated with 5 μM MGCD0103 for 4 days compared with fins treated with DMSO (Figure 4A), demonstrating that MGCD0103 effectively blocks Hdac1 activity in the caudal fin during regeneration. Furthermore, no alteration in general health was observed in fish incubated in MGCD0103-treated water for 10 days compared with animals incubated with DMSO-treated water (see Additional file 1: Figure S7).

Interestingly, treatment of regenerating fins with 5 μM MGCD0103 for 10 days resulted in a substantial reduction in regenerative growth (Figure 4B-E). However, the early stages of the regeneration process seemed not to be affected because wound healing was properly completed and a seemingly normal blastema was formed (Figure 4B,C), suggesting that Hdac1 activity is not essential for the earliest phases of regeneration. Regenerative outgrowth was impaired, starting from 3 dpa, and the regeneration process was progressively blocked and finally stopped (Figure 4D,E). Indeed, MGCD0103 treatment for 10 days resulted in the formation of abnormal curled fin-like structures, suggesting differentiation defects. To test whether Hdac1 inhibition also affects fin regeneration after blastema formation, fish were treated with MGCD0103 for 4 days starting at 3 dpa. As expected, we found that regenerative growth was blocked, similar to fins that were continuously treated from the time of amputation (see Additional file 1: Figure S8). This result confirmed that Hdac1 inhibition affects regeneration from the onset of regenerative outgrowth.

To test whether MGCD0103 treatment is reversible, fish were exposed to MGCD0103 for 10 days from the time of amputation, and then transferred to normal water for 10 additional days. In general, fins failed to restart the initially blocked regenerative process properly, indicating that the effects of Hdac1 inhibition on caudal fin regeneration are irreversible (see Additional file 1: Figure S9). However, occasionally, a few rays resumed regrowth (see Additional file 1: Figure S9), suggesting that some residual blastema cells retained their original regenerative potential despite the prolonged inhibition of regeneration.

Taken together, these data indicate that MGCD0103-mediated inhibition of Hdac1 does not affect wound healing and initial blastema formation, but impairs progression of fin regeneration during the regenerative outgrowth phase.

### The NuRD components *hdac1*, *chd4a*, *mta2*, and *rbb4* are required for blastema cell proliferation during the regenerative outgrowth phase

**Figure 5:**
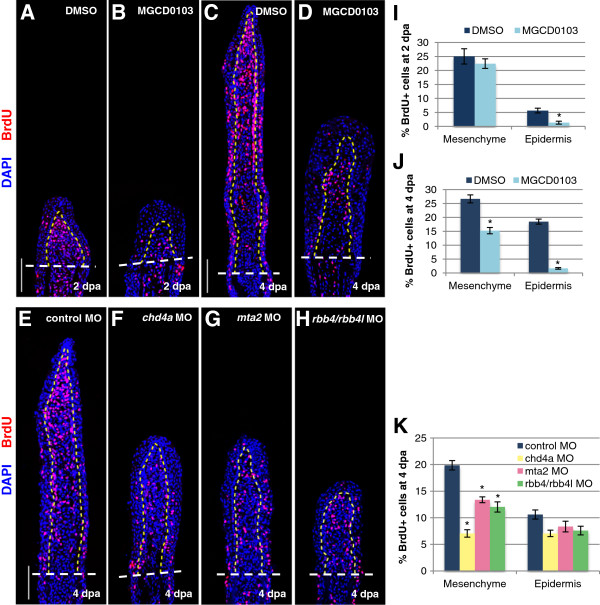
**The NuRD components *hdac1*, *chd4a*, *mta2*, and *rbb4 *are required for blastema cell proliferation during the regenerative outgrowth phase. (A-D)** Longitudinal sections of fin regenerates treated with DMSO or MGCD0103 at 2 **(A-B)** or 4 dpa **(C-D)** stained with BrdU antibody (red) and DAPI (blue). **(E-H)** Longitudinal sections of fin regenerates at 4 dpa injected with control **(E)**, *chd4a ***(F)**, *mta2 ***(G)**, or *rbb4* + *rbb4l ***(H)** MOs stained with BrdU antibody (red) and DAPI (blue). Yellow dashed lines indicate the boundary between the blastema and the wound epidermis, and white dashed lines indicate the amputation plane. Scale bars: 100 μm. **(I-K)** Percentage of BrdU-positive cells relative to the total number of cells (DAPI-labeled) in fin regenerates treated with DMSO or MGCD0103 at 2 dpa **(I)** or 4 dpa **(J)** or in fin regenerates injected with control, *chd4a*, *mta2*, or *rbb4* + *rbb4l* MOs **(K)**. Error bars represent the SEM. n = 9. *P < 0.01.

To understand the cause of the regenerative failure in fins deficient in these putative NuRD components, cell proliferation was assessed by labeling DNA-replicating cells with bromodeoxyuridine (BrdU) for 6 hours before fin collection. Because MGCD0103 treatment has the advantage of inhibiting Hdac1 from the time of amputation, cell proliferation was assessed in MGCD0103-treated fin regenerates during blastema formation and during regenerative outgrowth. At 2 dpa, mesenchymal cell proliferation was similar in DMSO-treated and in MGCD0103-treated fins, confirming that Hdac1 does not regulate blastema cell proliferation at this stage (Figure 5A,B,I). However, at 4 dpa, the percentage of BrdU-positive cells was significantly reduced in mesenchymal cells of MGCD0103-treated fish (Figure 5C,D,J). Consistently, cell proliferation was also significantly reduced in the blastema of fin regenerates injected with *chd4a*, *mta2*, or *rbb4/rbb4l* MOs at 4 dpa, that is, 24 hpi (Figure 5E-H,K). To determine whether the regenerative block was caused by cell death, activation of caspase-3 was examined by immunostaining to identify apoptotic cells. However, we did not observe any obvious increase in apoptosis in MGCD0103-treated or *chd4*, *mta2*, or *rbb4/rbb4l* MO-injected fin regenerates at 4 dpa (see Additional file 1: Figure S10; also data not shown). Altogether, these data suggest that inhibition of Hdac1 and morpholino-mediated knockdown of *chd4a*, *mta2*, and the two *rbb4* orthologs impair fin regeneration by reducing blastema cell proliferation during regenerative outgrowth, without inducing cell death.

MGCD0103 treatment resulted in a noticeable increase in wound epidermis (Figure 5B,D). However, no increase in cell proliferation was detected in the epidermis of MGCD0103-treated fins (Figure 5I,J). MGCD0103 treatment did not alter expression of the wound epidermis markers *wnt5b* and *lef1*, indicating that *hdac1* is not required for the correct specification of the wound epidermis (see Additional file 1: Figure S11). The enlargement of the epidermis in MGCD0103 regenerates could be the result of an abnormal migration of epithelial cells from the stump. As this phenotype was not observed in MO-injected fin regenerates, it is possible that Hdac1 plays an additional role independent of the Mi-2/NuRD complex during fin regeneration.

### Depletion of the NuRD components *hdac1*, *chd4a*, *mta2*, and *rbb4* results in abnormal patterning of actinotrichia during regeneration

**Figure 6:**
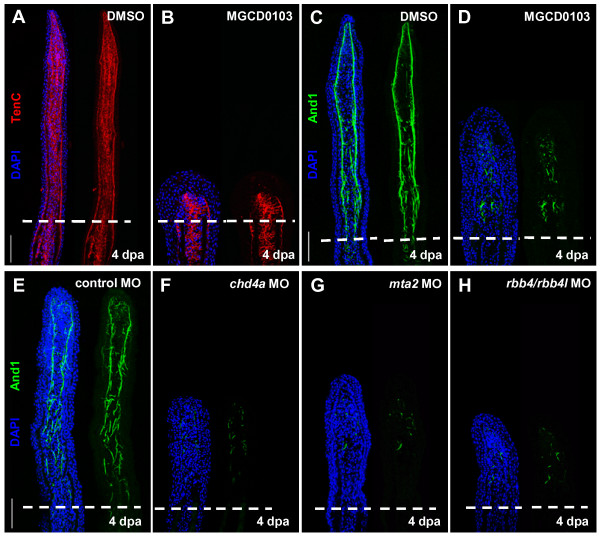
**Hdac1 inhibition and morpholino-mediated knockdown of *chd4a*, *mta2*, and the two *rbb4 *orthologs cause abnormal expression of Actinodin 1. (A-B)** Longitudinal sections of fin regenerates at 4 dpa treated with DMSO **(A)** or MGCD0103 **(B)** and stained with Tenascin C antibody (red) and DAPI (blue). Mesenchymal remodeling was not altered in MGCD0103-treated fins. **(C-H)** Longitudinal sections of fin regenerates at 4 dpa treated with DMSO **(C)** or MGCD0103 **(D)**, or injected with control **(E)**, *chd4a ***(F)**, *mta2 ***(G)**, or *rbb4* + *rbb4l ***(H)** MOs stained with Actinodin 1 antibody (green) and DAPI (blue). Depletion of the NuRD components *hdac1*, *chd4a*, *mta2*, or *rbb4/rbb4l* resulted in a disorganized expression pattern of Actinodin 1. Dashed lines indicate the amputation plane. Scale bars: 100 μm.

To examine the cellular consequences of NuRD component depletion, we assessed different cellular markers involved in fin regeneration. First, we examined mesenchymal reorganization by immunostaining with antibodies against Tenascin C, an extracellular matrix glycoprotein. Upon amputation, Tenascin C is rapidly induced in the mesenchyme below the amputation plane, and then expressed in the regenerating blastema [16,18]. We found that Tenascin C expression was normal in both MGCD0103-treated (Figure 6A,B) and *chd4a* MO-injected fins (see Additional file 1: Figure S12), suggesting that the *hdac1* and *chd4a* do not influence mesenchymal remodeling during blastema formation.

To evaluate the molecular specification of the blastema in fin regenerates deficient in NuRD components, we analyzed the expression of *msxb* by ISH*. msxb* is a molecular marker of the distal blastema and is required for blastema cell proliferation during fin regeneration [9]. We found that *msxb* transcripts were correctly expressed in MGCD0103-treated and in *chd4a* MO-injected fin regenerates (see Additional file 1: Figure S11), indicating that the distal blastema is correctly specified.

Finally, we analyzed the expression of Actinodin 1, a marker for actinotrichia-forming cells [53]. Actinotrichia are non-mineralized structural components that mechanically support the larval fin fold and the blastema of the fin regenerate [54,55]. The expression pattern of Actinodin 1 was completely disorganized at 4 dpa in fin regenerates treated with MGCD0103, compared with control fins (Figure 6C,D), indicating an abnormal patterning of actinotrichial fibers. A similarly disorganized expression pattern of Actinodin 1 was also observed in fins deficient in *chd4a*, *mta2*, or the two *rbb4* orthologs (Figure 6E-H). Altogether, these data suggest that depletion of the NuRD components results in cellular defects after the onset of regenerative outgrowth. Thus, these epigenetic factors are not essential for mesenchymal reorganization or initial blastema formation, but they are required for growth and correct patterning of the blastema during regenerative outgrowth.

### Hdac1 inhibition impairs osteoblast differentiation

**Figure 7:**
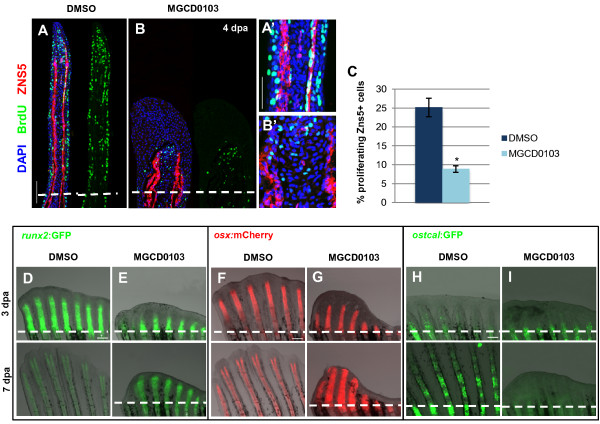
**Hdac1 is essential for redifferentiation of osteoblast during regeneration. (A-B)** Longitudinal sections of fin regenerates at 4 dpa treated with DMSO **(A)** or MGCD0103 **(B)** and triply stained with BrdU (green), ZNS5 antibody (red), and DAPI (blue). Dashed lines indicate the amputation plane. Proximal osteoblast nuclei acquired an elongated shape in control fins **(A′)**, whereas osteoblast nuclei rarely presented an elongated shape in MGCD0103-treated fins **(B′)**. **(C)** Percentage of ZNS5-positive cells at 4 dpa that were also positive for BrdU relative to the total number of ZNS5-positive cells in fin regenerates treated with DMSO or MGCD0103. Error bars represent the SEM. n = 15. *P < 0.01. **(D-I)** Caudal fins of *runx2:*GFP **(D-E)**, *osterix:*mcherry **(F-G)** or *osteocalcin:*GFP **(H-I)** transgenic fish treated with DMSO or MGCD0103 at 3 and 7 dpa. Constant exposure times were used. Dashed lines indicate the amputation plane. In DMSO-treated fish, the amputation plane is below the photographed part of the fin at 7 dpa. Scale bars: 100 μm **(A,D,F,H)**, 50 μm **(A′)**.

To further investigate the effect of Hdac1 inhibition during regeneration of the bony rays, we examined the progression of osteoblast differentiation during regenerative outgrowth. Osteoblasts are specialized cells that line the bony rays and secrete bone matrix. Upon fin amputation, mature osteoblasts dedifferentiate, re-enter the cell cycle, migrate distally in the blastema, and, during regenerative outgrowth, redifferentiate into osteoblasts in lateral regions of the blastema [12,56]. To assess osteoblast proliferation, osteoblasts were double-labeled at 4 dpa with BrdU and with Zns5, an antibody that marks osteoblasts at all stages of differentiation [57]. In control fins, BrdU-positive osteoblasts can be detected laterally in longitudinal fin sections (Figure 7A). Whereas nuclei of distally located proliferating osteoblasts are characterized by a spherical shape, proximal osteoblast nuclei begin to adopt an elongated shape, characteristic of their differentiated morphology. Interestingly, treatment of fins with 5 μM MGCD0103 resulted in a significant reduction in osteoblast proliferation, and the osteoblast nuclei rarely adopted an elongated shape (Figure 7A-C). Similar results were observed in *chd4a* MO-injected regenerating fins (see Additional file 1: Figure S13). Thus, the histone deacetylase Hdac1 is required for osteoblast proliferation and differentiation during regeneration, and the chromatin-remodeling protein Chd4a also seems to be involved in this process.

Next we used transgenic fish lines expressing fluorescent proteins to examine the expression of the bone differentiation markers *runx2*, *osterix*, and *osteocalcin*, which are sequentially activated during osteoblast differentiation [10,12]. In control fish, expression of the pre-osteoblast marker *runx2* and the intermediate osteoblast marker *osterix* is relatively low in unamputated fins, and it becomes strongly activated in the blastema during fin regeneration [12] (Figure 7D,F). In MGCD0103-treated fins, *runx2:*GFP and *osterix:*mCherry were both reactivated normally in the blastema at 3 dpa (Figure 7D-G), indicating that osteoblast dedifferentiation was not affected by Hdac1 inhibition. However, expression of *runx2* and *osterix* persisted in the proximal zone at 7 dpa, whereas it was progressively downregulated in the proximal differentiating zone in control fins (Figure 7D-G). This indicates a delay in the redifferentiation process in MGCD0103-treated fins.

The late bone differentiation marker *osteocalcin*, which labels mature osteoblasts, is downregulated in the stump of amputated fins and then robustly re-expressed in the proximal differentiated regenerate [12]. Interestingly, *osteocalcin:*GFP expression was not reactivated in fin regenerates treated with MGCD0103 at 7 dpa (Figure 7H-I). Furthermore, *osteocalcin:*GFP expression was also strongly reduced in the blastema of regenerating fins treated with MGCD0103, starting at 3 dpa, demonstrating that inhibiting Hdac1 after the blastema has been formed also blocks *osteocalcin* reactivation (see Additional file 1: Figure S14A). In uninjured fins, MGCD0103 treatment did not alter the expression of *osteocalcin:*GFP in mature bones (see Additional file 1: Figure S14B), indicating that Hdac1 inhibition specifically blocks the reactivation of *osteocalcin:GFP* expression in the differentiating blastema during fin regeneration. Taken together, our results indicate that Hdac1 inhibition prevents redifferentiation of osteoblast precursor cells. However, Hdac1 is not required for osteoblast dedifferentiation following fin amputation.

### Hdac1 inhibition results in the upregulation of regeneration marker and two pluripotency-associated genes

**Figure 8:**
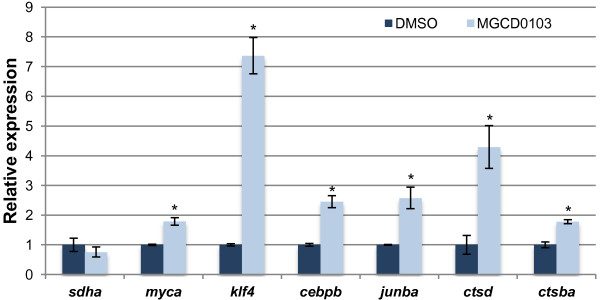
**Hdac1 regulates the expression of two pluripotency-associated genes and regeneration marker genes.** Quantitative real-time-PCR analyses of *sdha*, *myca*, *klf4*, *cebpb*, *junba*, *ctsd*, and *ctsba* mRNA in MGCD0103-treated fin regenerates relative to DMSO-treated fin regenerates at 4 dpa. The relative expression of the housekeeping gene *sdha* was not changed in MGCD0103-treated fin regenerates, indicating that Hdac1 inhibition does not cause a general increase in gene expression. Error bars represent the SEM. *P < 0.005.

In mammalian embryonic stem cells, the NuRD components HDAC1 and MBD3 have previously been shown to directly bind to and control the expression levels of pluripotency-associated factors [58,59]. Therefore, to determine whether Hdac1 also regulates expression of pluripotency-associated factors during regeneration, we measured the expression levels of several candidate genes by qRT-PCR following MGCD0103 treatment. We found that two pluripotency-associated genes, *myca* and *klf4*, were upregulated in MGCD0103-treated fin regenerates at 4 dpa (Figure 8). In addition, we found that MGCD0103 treatment also increased the expression levels of four genes involved in regeneration. *junba* encodes a transcription factor of the Junb family, which is immediately induced upon fin amputation and required for blastemal proliferation in zebrafish [44,60]. The two cathepsins *ctsba* and *ctsd* are proteases whose expression is upregulated during dedifferentiation in regenerating tissues [61,62]. *cebpb* encodes a bZIP transcription factor upregulated in regenerating liver and required for the proliferative response [63]. Thus, these data demonstrate that Hdac1 represses, directly or indirectly, the transcription of two factors associated with pluripotency, and of several regeneration markers associated with dedifferentiation during regenerative outgrowth.

## Discussion

Here we show evidence for the role of putative NuRD components during fin regeneration in zebrafish. We propose a model in which a specialized Mi-2/NuRD complex could be involved in blastema cell proliferation and redifferentiation during regenerative outgrowth. The zebrafish genome encodes orthologs for every subunit of the vertebrate NuRD complex. However, we found that transcripts of the putative NuRD components *chd4a*/Mi-2, *hdac1*/HDAC1/2, *rbb4*/RBB4/7, and *mta2*/MTA were specifically co-induced in the blastema during adult and embryonic fin regeneration, and displayed similar spatial and temporal expression patterns. Although there are several homologs for each NuRD component encoded by the genome of zebrafish (with the exception of *hdac1*), only one of each seems to be present in the putative NuRD complex involved in fin regeneration. Thus, the combinatorial assembly of the different paralogs of each NuRD subunit may define its specific function.

We also found that disruption of these putative 'regenerating' NuRD components impaired fin regeneration. Chemical inhibition of Hdac1 by MGCD0103 and morpholino-mediated knockdown of *chd4a*, *mta2*, and the two *rbb4* orthologs resulted in the reduction in blastema cell proliferation during regenerative outgrowth. However, these putative NuRD components seem not to be required for the earliest stages of fin regeneration. This is demonstrated by the facts that inhibition of Hdac1 starting from the time of amputation had no influence on wound healing and blastema formation. In addition, Tenascin C, an early mesenchymal marker, and *msxb*, a marker of the distal blastema, were normally expressed in *chd4a*-deficient and *hdac1*-deficient fin regenerates.

The wound epidermis was noticeably enlarged in *hdac1*-deficient fin regenerates. It is likely that the increase in the epidermis size resulted from the migration of epithelial cells from the stump, as no increase in cell proliferation was detected in the wound epidermis of MGCD0103-treated fins. Although Hdac1 inhibition reduced cell proliferation in the blastema, epithelial cells might continue to migrate and accumulate, forming an enlarged wound epidermis. This phenotype was not observed in fins deficient in the other NuRD components *chd4a*, *mta2*, and *rbb4*. As HDAC1 is also known to be a catalytic subunit of other multiprotein complexes in mammals, such as CoREST and Sin3 complexes [64], we cannot exclude that Hdac1 plays additional roles independent of the NuRD complex during fin regeneration. Further experiments are needed to identify direct interacting partners of these proteins in regenerating fins.

We found that on addition to the proliferation defects of blastema cells during regenerative outgrowth, Hdac1 inhibition and knockdown of *chd4a*, *mta2*, and the two *rbb4* orthologs resulted in an abnormal expression pattern of Actinodin 1, a component of structural fibers called actinotrichia. During development, actinotrichia support the fragile fin fold of the larvae. During regeneration, actinotrichia are formed between the epidermis and the blastema prior to lepidotrichia regrowth, and are probably required for shaping the regenerate [53,55]. Consistently, osteoblast proliferation and differentiation were also impaired in *hdac1*-deficient fin regenerates. Analysis of the bone differentiation markers *runx2*, *osterix*, and *osteocalcin,* which are sequentially expressed during fin regeneration [12], indicated that Hdac1 inhibition did not interfere with osteoblast dedifferentiation. However, expression of the late bone differentiation marker *osteocalcin*, expressed only in mature bones, was not reactivated in the redifferentiating proximal fin regenerates after Hdac1 inhibition, suggesting that Hdac1 is essential for redifferentiation of osteoblast precursor cells. Indeed, expression of *runx2* and *osterix* persisted in the proximal blastema of MGCD0103-treated fins, indicating that blastema cells were blocked in an intermediate state.

The effects of morpholino-mediated knockdown of the other NuRD components were not persistent, and regeneration resumed 48 hours post-injection. Morpholino injection has some limitations and is not an appropriate technique to analyze differentiation defects of bone-forming cells. Therefore, we were not able to analyze the consequences of morpholino-mediated knockdown of *chd4a*, *mta2*, and *rbb4* on osteoblast regeneration.

Somewhat reminiscent to our findings in zebrafish, the planarian ortholog Smed-CHD4 is also essential for regeneration and neoblast differentiation in *Schmidtea mediterranea*[41]. Smed-CHD4 expression is induced in neoblasts after wounding, and CHD4(RNAi) worms fail to regenerate following amputation or even to maintain normal tissue turnover. In CHD4-depleted animals, the number of neoblast progeny cells is reduced because neoblasts are unable to produce progeny cells committed to differentiation [41]. It is, however, not clear whether Smed-CHD4 also acts as a member of a NuRD complex.

Recently, an elegant model has been proposed in which the NuRD complex binds to the promoters of numerous pluripotency genes in embryonic stem cells (ESCs), probably to fine-tune the transcription levels of the genes and to maintain the differentiation responsiveness of the ESCs [59]. In the absence of a functional NuRD complex, expression of these genes is increased above a threshold, thereby blocking the response of ESCs to developmental cues and preventing them from exiting from the self-renewal state [65].

We hypothesize that the Mi-2/NuRD complex might have a similar function during fin regeneration in zebrafish. This is suggested by our findings that the NuRD components were all expressed in the proliferative zone of the blastema during regenerative outgrowth and that their depletion resulted in a reduction in blastema proliferation and an increase in cellular differentiation defects. In addition, Hdac1 inhibition leads to the upregulation of the two pluripotency-associated genes, *myca* and *klf4*, and genes encoding regeneration markers associated with dedifferentiation. The histone deacetylase Hdac1 might be required to downregulate the expression of these genes, thereby promoting the responsiveness of blastema cells to regenerative signals in order to ensure correct reconstitution of lost tissues. In the absence of Hdac1, expression of these genes continues to be high, resulting in the blocking of blastema cells in an undifferentiated or partially differentiated state. Further experiments are needed to determine whether Hdac1 represses the expression of these genes in a NuRD-dependent context.

Epigenetic mechanisms are critical for the regulation of gene expression and lineage specification during development [66]. A previous study has shown that H3K27me3 demethylase is required for caudal fin regeneration in zebrafish [67]. Stewart *et al*. established that many developmental regulatory genes involved in fin regeneration are poised in a bivalent H3K4me3/H3K27me3 chromatin domain, and that the demethylation of H3K27me3 enables activation of expression of these genes in response to injury. It is possible that the zebrafish maintains key developmental regulatory genes in a dormant state to allow rapid switching of their expression profile through epigenetic mechanisms in response to amputation.

## Conclusion

Our study provides further *in vivo* evidence for the involvement of key epigenetic factors in epimorphic fin regeneration in zebrafish. We propose a model in which a specialized Mi-2/NuRD complex is induced in the blastema of regenerating fins to coordinate proliferation and differentiation and thus reform the missing tissues. Even though different animals may be endowed with different regenerative capacities, crucial regeneration markers are conserved in all vertebrate species. Thus, fin regeneration constitutes an excellent *in vivo* system to study the epigenetic mechanisms regulating regeneration, and to elucidate how this process is maintained in some vertebrates.

## Methods

The experimental animal research was approved by the cantonal veterinary office of Fribourg (Switzerland).

### Zebrafish and fin amputation

The following zebrafish strains were used in this study: AB wild-type strain, and the *osterix:*mCherry (*OlSp7*:mCherry^zf131^) [68], *runx2*:GFP (Has.RUNX2-Mmu.Fos:EGFP^zf259^), and *osteocalcin*:GFP (*Ola.Osteocalcin.1*:EGFP^hu4008^) fish lines [12]. 6-24 month-old adult fish were anesthetized in 0.1% tricaine, and the caudal fins were amputated with a razor blade. Animals were allowed to regenerate at 28.5°C. Larval fin folds were amputated as previously described [43]. Larvae were allowed to regenerate at 28.5°C and were collected at 1 dpa for further analysis. For proliferation assays, fish were incubated for 6 hours before fin collection in fish water containing 50 μg/ml BrdU (Sigma-Aldrich, Buchs, Switzerland).

### MGCD0103 treatment

MGCD0103 (Selleckchem, Houston, USA) was dissolved in DMSO at 10 mM stock concentration and then added to the fish water at a final concentration of 5 μM. 0.05% DMSO was added to the water of control fish.

### Morpholino knockdown

The following antisense vivo-morpholinos (Gene Tools, OR, USA) were used: *chd4a* translational blocking (MOTL) 5′-CTCTATCGTCCTCACTGCCGGACAT-3′, *chd4a* splice blocking (MOSP; targeting the exon 8–intron 8 boundary) 5′-AAAGAGAGTGAGATCCTCACCCTTT-3′, *rbb4* MOTL 5′- ACACTTCTTTATCGGCCATTTTGGC-3′, *rbb4l* MOTL 5′- ATGCAGCTTCTTTATCAGCCATAAC-3′ and *mta2* MOTL 5′- CCGCCATTCTCTCGCTCTCCTAAAC-3′. The standard vivo-morpholino from Gene Tools (5′-CCTCTTACCTCAGTTACAATTTATA-3′) was used as negative control. MOs were injected as described previously [16] with a FemtoJet microinjector (Eppendorf) into the dorsal half of regenerating fins at 3 dpa. The ventral half of the fins was uninjected, and was used as an internal control. Fins were photographed immediately after injection and at 24 hours post-injection with a stereomicroscope (MZ16) and camera (DFC480) (both Leica). The percentage of regeneration was calculated as previously described [18,26]. Areas of the dorsal and the ventral half of regenerating fins were measured with Image J 1.43 q software. The percentage of regeneration was calculated with the following formula:

$$\left(D_{4\;\mathrm{dpa}}‒D_{3\;\mathrm{dpa}}\right)/\left(V_{4\;\mathrm{dpa}}‒D_{3\;\mathrm{dpa}}\right)\times 100,$$

where D is the area of the dorsal side and V is the area of the ventral side of the fin regenerate. Statistical significance was determined with the Student’s t-test, and significance was set at *P* < 0.01.

### *In situ* hybridization

Whole-mount *in situ* hybridization and *in situ* hybridization on fin cryosections was performed as previously described [22,69]. Normarski imaging was performed with a Zeiss Axioplan microscope. The following primers were used to generate ISH probes:

*chd4a* (NM_001044858.1): F GTTCCCAAAGCAGAAGATGC, R TTCGTTAAGAATGGCGAACC (735 bp),

*chd4b* (XM_680607.5): F GGTGAAAGGCTCCAGACAG, R GCGGCTCTCTCTTCATTCTG (513 bp),

*chd3* (XM_691549.5): F CTGACAAGACGGAGAAGAGC, R CCTGAAAGCAGCCAGAAGTC (730 bp),

*hdac1* (NM_173236.1): F CATTAACTGGGCAGGAGGTC, R GGCTATCCGCTTATCGTGAG (847 bp),

*mta2* (NM_214695.1): F CAACCAGATCACAGCACCTG, R CCACAAACACCACAGGATTG (791 bp),

*rbb4* (NM_212595.1)**:** F ATTTGGTGGTTTTGGCTCAG, R CCCATGGTTCATTTGGATTC (854 bp),

*wnt5b* (NM_130937.1): F CAAGTGTCATGGCGTCTCAG, R CAACAGCAAGGTGGAGTGTG (850 bp)

*lef1* (NM_131426.1): F ATGCACGCTGAAGGAGAG, R GAACCCAAGATGTCGAGGAG (801 bp)

*msxb* (NM_131260): F GAGAATGGGACATGGTCAGG, R GCGGTTCCTCAGGATAATAAC (721 bp)

### Immunohistochemistry

Fins were fixed in 4% paraformaldehyde in PBS, embedded in OCT, and cryosectioned. Antibody staining was performed as previously described [22]. The following primary antibodies were used: rat anti-BrdU (1:200), rabbit anti-active-caspase 3 (1:10000) (both Abcam), rabbit anti-Tenascin C (1:500; US Biological), mouse anti-Zns5 (1:100; Zebrafish International Resource Center), rabbit anti-And1 (1:5000; Eurogentec). The following secondary antibodies were used at a concentration of 1:500: goat anti-rat Alexa Fluor 488 (Molecular Probes), and goat anti-rabbit Cy3-conjugated and anti-mouse Cy5-conjugated antibodies (Jackson ImmunoResearch).

For proliferation assay, BrdU-positive cells distal to the amputation plane were counted in the mesenchyme and epidermis, and the number of BrdU-positive cells was normalized to the total number of DAPI-stained nuclei. Fluorescent pictures were taken with a confocal microscope (TCS SP5; Leica) and Image J 1.43q software was used for the measurements.

### Quantitative real-time PCR

Fin regenerates were collected and total RNA was extracted using Qiazol (Qiagen, Basel, Switzerland). cDNA was synthesized using the QuantiTect Reverse Transcription Kit (Qiagen, Basel, Switzerland). Quantitative real-time PCR was performed in triplicate using the SensiMix SYBR No-ROX Kit (Bioline, Luckenwalde, Germany). Relative expression levels were normalized to *β-actin* levels. At least two independent experiments were performed for each target, and data were pooled to generate mean normalized RNA levels. The following primers were used for qPCR experiments:

*bactin1* (NM_131031.1): F ACATCAGGGAGTGATGGTTG, R TCACAATACCGTGCTCAATG,

*chd4a* (NM_001044858.1): F GAGAAAGTGCCAAAGACAGC, R AATTCGGTGAATCCTCCATC,

*chd4b* (XM_680607.5): F CATGGGAGACGATATCGAAG, R CGTTTGCTAGTCCTGCTTTC,

*chd3* (XM_691549.5): F ACATCCCTGAGTTTGCTCTG, R CGTTCTCCTCTCTCCTCCTC,

*hdac1* (NM_173236.1): F TGACAAACGCATCTCCATTC, R TCTTCACTCGTTTTGGCTTC

*mta1* (XM_001333237.3): F CGTACACACCTGTCAACACC, R TGCGCCTCGAGATATCTAAC

*mta2* (NM_214695.1): F AAAGATTTGGCCATTCAAGC, R AAATGACCTCCAGCATTGTC

*mta3* (NM_199912.2): F CTGCACCTAACGAATCACG, R GTCTTCATGGAGGATTTTGG

*rbb4* (NM_212595.1): F TATCCATGGAGGCCATACAG, R TAGATGTTCTCCGCCATCTG

*rbb4l* (NM_212610.1): F AAGTATGGCAGATGGCTGAG, R TGTGAAGAGTAAGAAGGGGTTG

*mbd3a* (NM_212769.1): F AGACATGCTGGCACACATC, R GTTCAGCCTCTCATCTGATTG

*mbd3b* (NM_212580.1): F AGCACAGGTATTTAGATGTGTCTG, R GCTAATCTGGGAGATGAAAATG

*mbd2* (NM_212768.1): F CTGCAAAGCGTTCAGTGTTAC, R GCCTGTGGGATCTCTCTAAAC

*klf4* (NM_131723.1): F GACGCACACAGGTGAGAAG, R GTCCGGTGTGTTTCCTGTAG

*myca* (NM_131412.1): F GGCAGCGATTCAGAAGATG, R CTTTTCTGTCGCTTTTCCAC

*cebpb* (NM_131884.2): F GACGCGAGAGGAACAATCTC, R GCTTCTGTAACCGGTCGTTC

*ctsd* (NM_131710.1): F CATCGGCAGTGGACTATCTC, R CCATGTACTCTCCCTGCATC

*ctsba* (NM_213336.2): F TTTGGGAAGACGTCCTACAG, R AGCAGGAAATCCTCATAGACC

*junba* (NM_213556.3): F AGTACCACCACCATCACCAC, R GTCTGTGGCTCCTCTTTCAG

*sdha* (NM_200910.1)**:** F TGTGTGGAACACTGATCTGG, R TCCACACGATCCTTGAAGTC

For MOSP efficacy, segments of the correctly spliced *chd4a* mRNA around the exon 8 were amplified with the following primers:

prCP46: F TCCTTATCGTGACAGGCCTAC

prCP47: R GGAGTAGGGCCCTTTCAATC

prCP82: R AAGCAGACCATGTGATAGGC

### Western blot

Fin regenerates were disrupted using glass beads in a mixture of 240 mM Tris HCl pH 6.8, 8% SDS, 40% glycerol, 0.01% bromophenol blue, and 1.4 M β-mercaptoethanol. Then 20 μg of total proteins were loaded per lane and separated by SDS-PAGE (12%). Even loading was verified by staining with Ponceau S and with β-actin antibodies (1:2000; Sigma). Proteins were transferred onto nitrocellulose membranes, and blots were incubated in 5% milk with rabbit anti-Histone H3 (1:2000), rabbit anti-acetyl-histone H3 (1:1000), anti-acetyl-histone H4 (1:1000) (all Millipore) and β-actin (1:2000; Sigma). Secondary HRP anti-rabbit and anti-mouse antibodies (Sigma) were used at 1:10,000.

## Abbreviations

And1: Actinodin 1; BrdU: 5-bromo-2′-deoxyuridine; DAPI: 4′,6-diamidino-2-phenylindole; DMSO: Dimethyl sulfoxide; dpa: Day post-amputation; hpa: Hour post-amputation; MO: Vivo-morpholino; MOSP: Splice blocking vivo-morpholino; MOTL: Translational blocking vivo-morpholino; qRT-PCR: quantitative real-time polymerase chain reaction; TenC: Tenascin C.

## Competing interests

The authors declare no competing interests.

## Authors’ contributions

FM, CW, and AJ conceived and planned the study. CP performed the experiments. CP, FM, CW, and AJ wrote the manuscript. All the authors discussed the results and commented on the manuscript. All authors read and approved the final manuscript.

## Supplementary Material

Additional file 1: Figure S1 The genome of zebrafish encodes three Mi-2 orthologs. **Figure S2.** One of the three Mi-2 homologs, *chd4a,* is induced during regeneration in the adult caudal fin. **Figure S3. ***chd4a* is expressed during regenerative outgrowth in adult caudal fin. **Figure S4. ***chd4a*, *chd4b*, and *chd3* are expressed in developing zebrafish embryos. **Figure S5.** The splice blocking antisense *chd4a* MOSP efficiently impairs the splicing of *chd4a* transcript. **Figure S6.** Zebrafish Hdac1 and human HDAC1/HDAC2 are highly conserved. **Figure S7.** MGCD0103 treatment does not affect the general health of zebrafish. **Figure S8.** Hdac1 inhibition after blastema formation is sufficient to impair regenerative outgrowth. **Figure S9.** The effects of MGCD0103 treatment are not reversible. **Figure S10.** Hdac1 inhibition and *chd4a* knockdown do not result in the activation of the apoptosis marker caspase-3. **Figure S11. ***wnt5b*, *lef1*, and *msxb* are expressed in *chd4a* morpholino oligonucleotide (MO)-injected and MGCD0103-treated fins. **Figure S12. ***chd4a* knockdown does not affect Tenascin C expression. **Figure S13. ***chd4a* knockdown reduces osteoblast proliferation. **Figure S14.** Hdac1 inhibition after blastema formation is sufficient to block reactivation of *osteocalcin* expression. Click here for file
