# Supplementary material for: Specific NuRD components are required for fin regeneration in zebrafish
Source: BMC Biol. 2014 Apr 29;12:30. doi: 10.1186/1741-7007-12-30 (PMC4038851; doi:10.1186/1741-7007-12-30)
Supplement: Additional file 1: Figure S1 — The genome of zebrafish encodes three Mi-2 orthologs. Figure S2. One of the three Mi-2 homologs, chd4a, is induced during regeneration in the adult caudal fin. Figure S3. chd4a is expressed during regenerative outgrowth in adult caudal fin. Figure S4. chd4a, chd4b, and chd3 are expressed in developing zebrafish embryos. Figure S5. The splice blocking antisense chd4a MOSP efficiently impairs the splicing of chd4a transcript. Figure S6. Zebrafish Hdac1 and human HDAC1/HDAC2 are highly conserved. Figure S7. MGCD0103 treatment does not affect the general health of zebrafish. Figure S8. Hdac1 inhibition after blastema formation is sufficient to impair regenerative outgrowth. Figure S9. The effects of MGCD0103 treatment are not reversible. Figure S10. Hdac1 inhibition and chd4a knockdown do not result in the activation of the apoptosis marker caspase-3. Figure S11. wnt5b, lef1, and msxb are expressed in chd4a morpholino oligonucleotide (MO)-injected and MGCD0103-treated fins. Figure S12. chd4a knockdown does not affect Tenascin C expression. Figure S13. chd4a knockdown reduces osteoblast proliferation. Figure S14. Hdac1 inhibition after blastema formation is sufficient to block reactivation of osteocalcin expression. [file 1741-7007-12-30-S1.pdf]

Supplementary figures

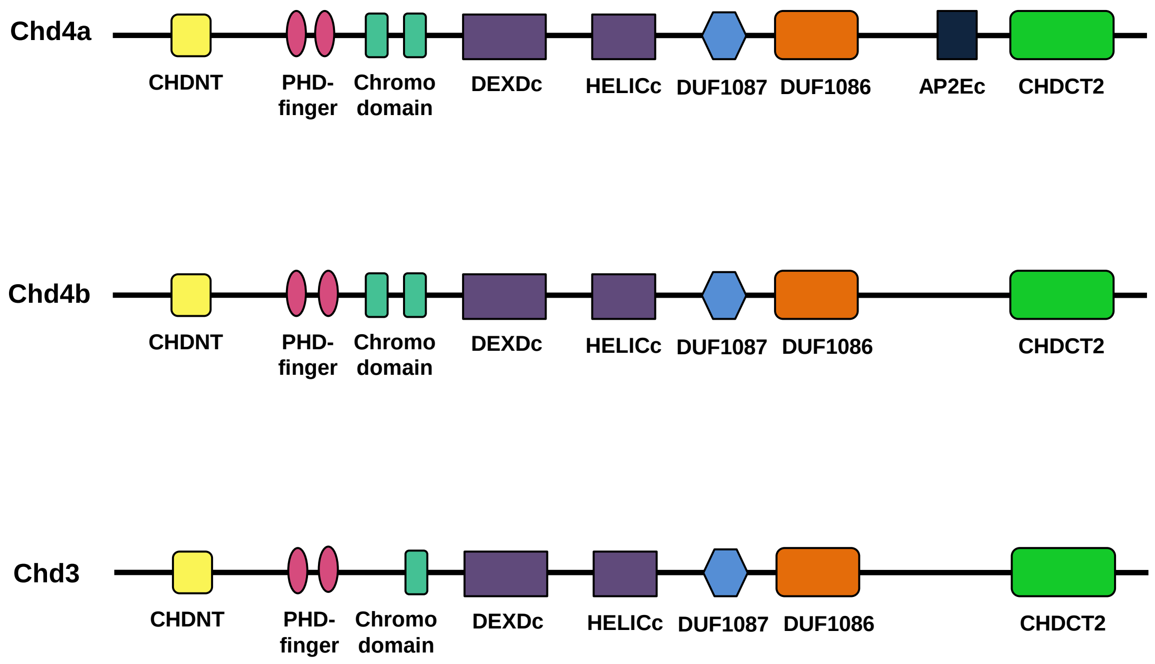

Figure S1. The genome of zebrafish encodes three Mi-2 orthologues.

Schematic representation of zebrafish Mi-2 proteins, Chd4a (NP_001038323.1), Chd4b (XP_685699.4) and Chd3 (XP_696641.3). Conserved domains determined with the Conserved Domain Database (NCBI) are shown.

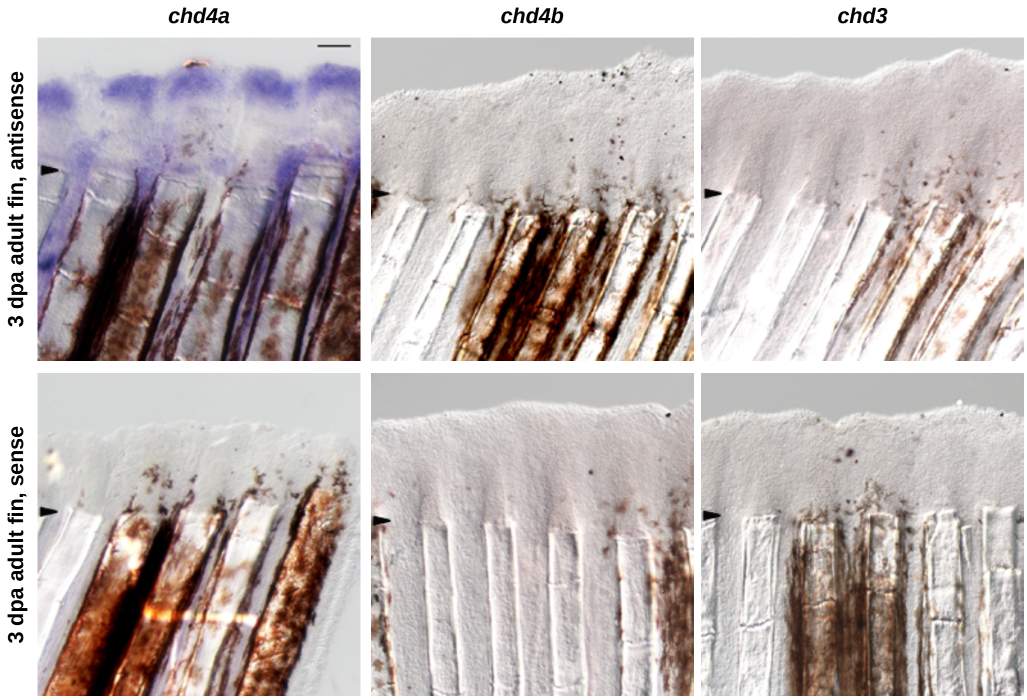

Figure S2. One of the three Mi-2 homologues, *chd4a,* is induced during regeneration in the adult caudal fin.

In situ hybridization with *chd4a*, *chd4b*, and *chd3* mRNA antisense probes on whole regenerating adult caudal fins at 3 dpa. No signals were detected with the corresponding sense RNA probes. Arrowheads indicate the amputation plane. Scale bar: 100 µm.

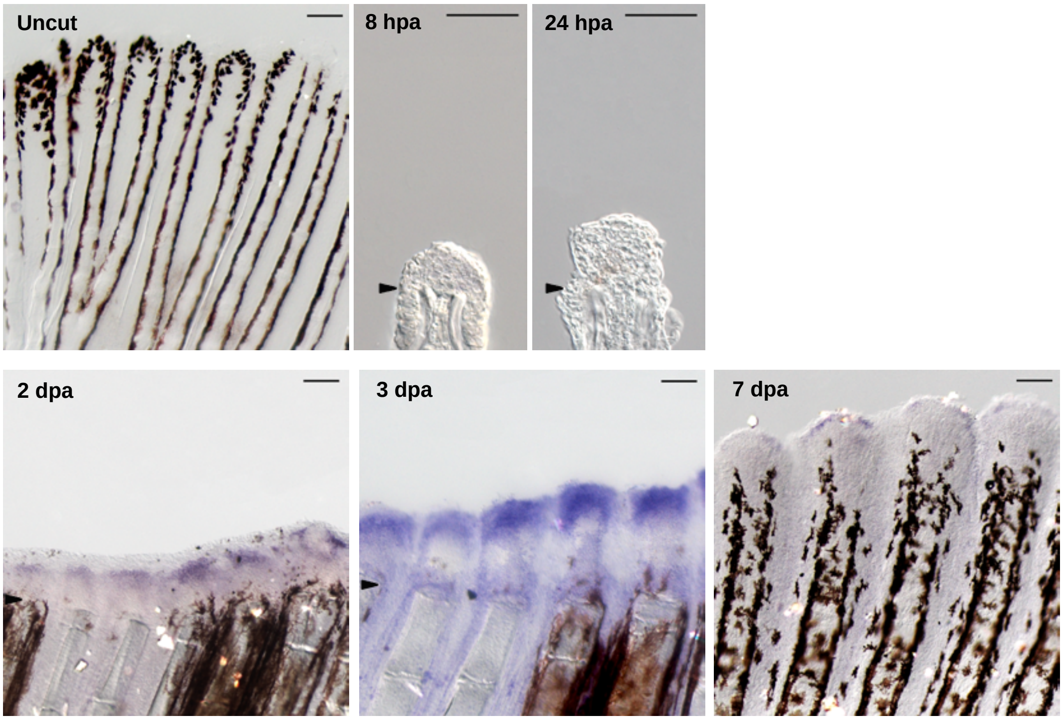

Figure S3. ***chd4a*** **is expressed during regenerative outgrowth in adult caudal fin.**

In situ hybridization with *chd4a* mRNA antisense probe on cryosections at 8 hpa and 24 hpa and on whole fins at 2 dpa, 3 dpa, 7 dpa and in uninjured fin. *chd4a* expression was first observed at 2 dpa with a robust signal at 3 dpa. Arrowheads indicate the amputation plane. Scale bars: 100 µm.

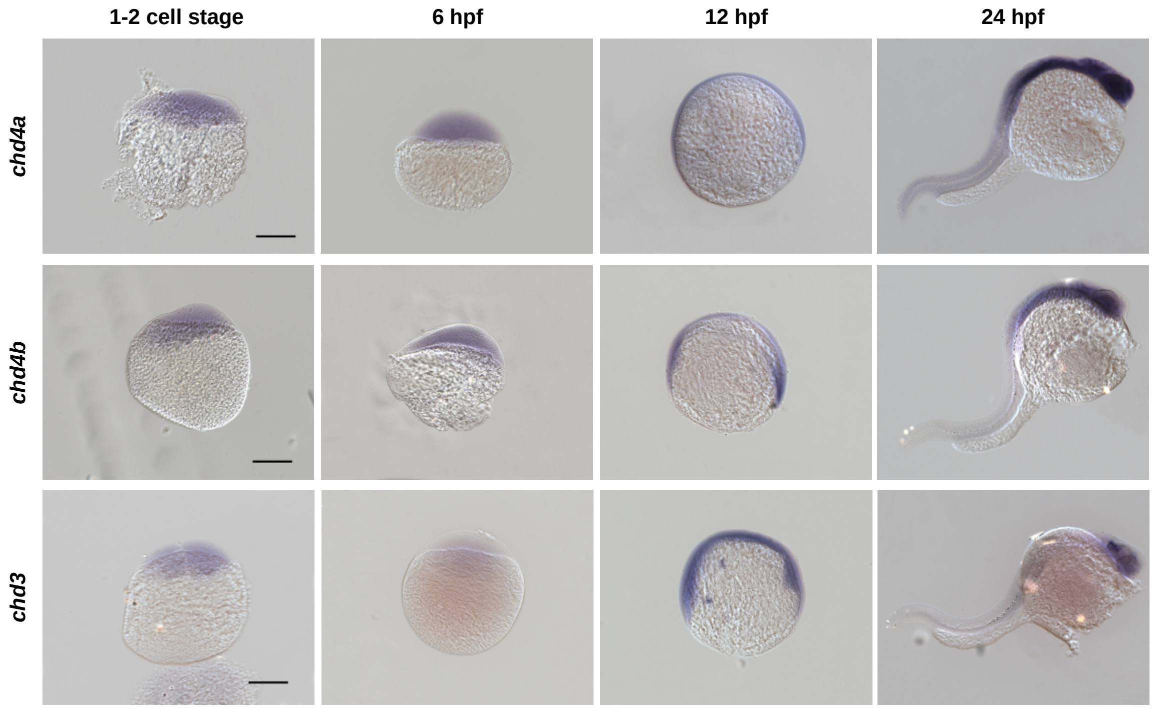

Figure S4. *chd4a*, *chd4b* and *chd3* are expressed in developing zebrafish embryos.

Whole-mount *in situ* hybridization with *chd4a, chd4b* and *chd3* RNA antisense probes in zebrafish embryos at 1-2 cell stage, 6 hours post-fertilization (hpf), 12 hpf and 24 hpf. The expression pattern of *chd4a*, *chd4b* and *chd3* in embryos suggest that they might also play a role during embryonic development. Scale bars: 50 µm.

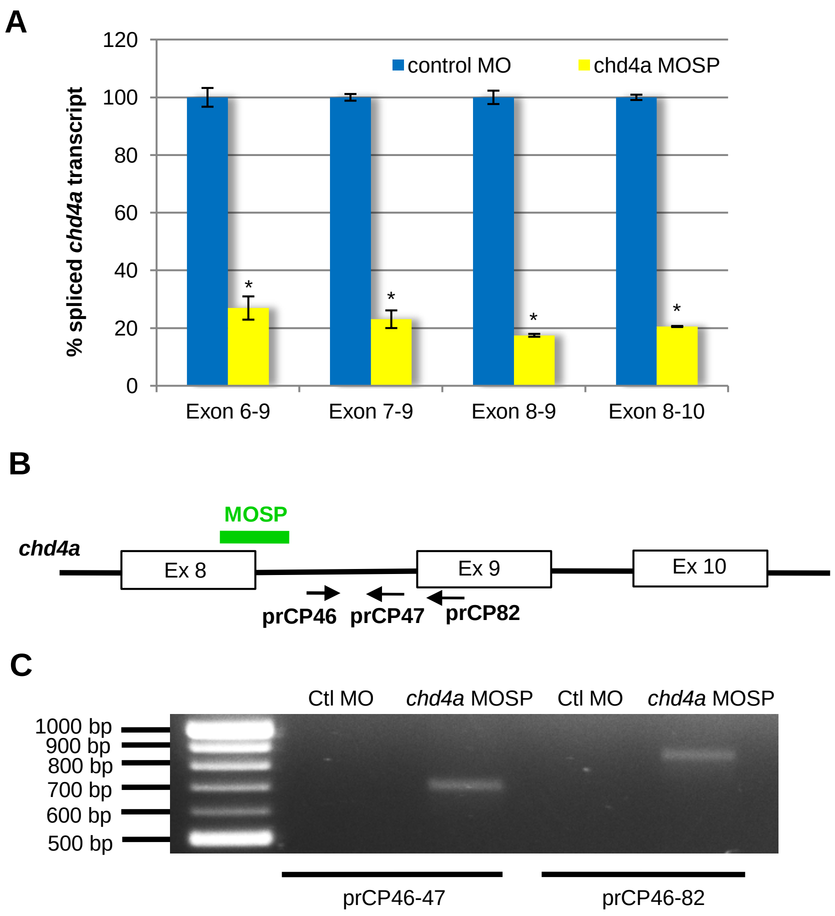

Figure S5. The splice blocking antisense *chd4a* MOSP efficiently impairs the splicing of *chd4a* transcript.

The splice-blocking *chd4a* MOSP was predicted to target the exon 8 - intron 8 boundary leading to an aberrant splicing of *chd4a* transcript. (A) The level of correctly spliced *chd4a* mRNA was determined by quantitative RT-PCR in morphant embryos. The control MO or the splice blocking antisense *chd4a* MOSP was injected into 1-2 cell stage embryos and RNA from 24 hpi embryos was extracted. The level of correctly spliced *chd4a* mRNA was significantly reduced in *chd4a* morphant embryos compared to control embryos, indicating that the splicing of *chd4a* transcript was impaired. Error bars represent the s.e.m. * $P<0.001$ (B) Unprocessed *chd4a* transcript is schematically represented. The primers prCP46, prCP47 and prCP82 used to amplified segments of the incorrectly spliced *chd4a* RNA are indicated. (C) Segments of the intron 8 of *chd4a* transcript were amplified by PCR with the indicated primers from cDNA synthetized from RNA of control embryos or *chd4a* MOSP morphant embryos.

```
                        Deacetylase domain
zHdac1    MALSSQGTKKKVCYYYDGDVGNYYYGQGHPMKPHRIRMTHNLLLNYGLYRKMEIYRPHKA 60
hHDAC1    MAQT-QGTRRKVCYYYDGDVGNYYYGQGHPMKPHRIRMTHNLLLNYGLYRKMEIYRPHKA 59
hHDAC2    MAYSQGGGKKKVCYYYDGDIGNYYYGQGHPMKPHRIRMTHNLLLNYGLYRKMEIYRPHKA 60
          **  :  *  ::*********:**************************************

zHdac1    NAEEMTKYHSDDYIKFLRSIRPDNMSEYSKQMQRFNVGEDCPVFDGLFEFCQLSTGGSVA 120
hHDAC1    NAEEMTKYHSDDYIKFLRSIRPDNMSEYSKQMQRFNVGEDCPVFDGLFEFCQLSTGGSVA 119
hHDAC2    TAEEMTKYHSDEYIKFLRSIRPDNMSEYSKQMQRFNVGEDCPVFDGLFEFCQLSTGGSVA 120
          .**********:************************************************
                                                              Zn2+Zn2+
                                                              ●  ●
zHdac1    GAVKLNKQQTDIAINWAGGLHHAKKSEASGFCYVNDIVLAILELLKYHQRVLYIDIDIHH 180
hHDAC1    SAVKLNKQQTDIAVNWAGGLHHAKKSEASGFCYVNDIVLAILELLKYHQRVLYIDIDIHH 179
hHDAC2    GAVKLNRQQTDMAVNWAGGLHHAKKSEASGFCYVNDIVLAILELLKYHQRVLYIDIDIHH 180
          .*****:****:*:**********************************************

zHdac1    GDGVEEAFYTTDRVMTVSFHKYGEYFPGTGDLRDIGAGKGKYYAVNYPLRDGIDDESYEA 240
hHDAC1    GDGVEEAFYTTDRVMTVSFHKYGEYFPGTGDLRDIGAGKGKYYAVNYPLRDGIDDESYEA 239
hHDAC2    GDGVEEAFYTTDRVMTVSFHKYGEYFPGTGDLRDIGAGKGKYYAVNFPMRDGIDDESYGQ 240
          **********************************************:*:*********
                                 Zn2+
                                 ●
zHdac1    IFKPIMSKVMEMYQPSAVVLQCGADSLSGDRLGCFNLTIKGHAKCVEYMKSFNLPLLMLG 300
hHDAC1    IFKPVMSKVMEMFQPSAVVLQCGSDSLSGDRLGCFNLTIKGHAKCVEFVKSFNLPMLMLG 299
hHDAC2    IFKPIISKVMEMYQPSAVVLQCGADSLSGDRLGCFNLTVKGHAKCVEVVKTFNLPLLMLG 300
          ****::******:**********:**************:********  :*:****:****

zHdac1    GGGYTIKNVARCWTFETAVALDSTIPNELPYNDYFEYFGPDFKLHISPFNMTNQNTNDYL 360
hHDAC1    GGGYTIRNVARCWTYETAVALDTEIPNELPYNDYFEYFGPDFKLHISPSNMTNQNTNEYL 359
hHDAC2    GGGYTIRNVARCWTYETAVALDCEIPNELPYNDYFEYFGPDFKLHISPSNMTNQNTPEYM 360
          ******:*******:*******  ************************ *******  :*:

zHdac1    EKIKQRLFENLRMLPHAPGVQMQAIPEDAVQEDSGDE-EDDPDKRISIRAHDKRIACDEE 419
hHDAC1    EKIKQRLFENLRMLPHAPGVQMQAIPEDAIPEESGDEDEDDPDKRISICSSDKRIACEEE 419
hHDAC2    EKIKQRLFENLRMLPHAPGVQMQAIPEDAVHEDSGDEDGEDPDKRISIRASDKRIACDEE 420
          *****************************: *:****  :********* : ******:**

zHdac1    FSDSEDEGQGGRRNAANYKK-PKRVKTEEEK--DGEEKKDVKEEEKASE---EKMDTKGP 473
hHDAC1    FSDSEEEGEGGRKNSSNFKK-AKRVKTEDEKEKDPEEKKEVTEEEKTKE---EKPEAKGV 475
hHDAC2    FSDSEDEGEGGRRNVADHKKGAKKARIEEDKKETEDKKTDVKEEDKSKDNSGEKTDTKGT 480
          *****:**:***:* ::.** .*:.: *::*    ::*.:*.**:*:.:    ** ::**

zHdac1    KEELKTV- 480
hHDAC1    KEEVKLA- 482
hHDAC2    KSEQLSNP 488
          *.*
```

Zn2+ ● in contact with zinc ligand

Figure S6. Zebrafish Hdac1 and human HDAC1/HDAC2 are highly conserved

Amino acid sequence alignment of zebrafish Hdac1 (NP_775343), human HDAC1 (NP_004955) and human HDAC2 (NP_001518). The catalytic domain (highlighted in red) of the three proteins is highly conserved (93% identity). The active center of HDAC1/2 contains a zinc ion, that binds to three conserved residues (indicated in blue).

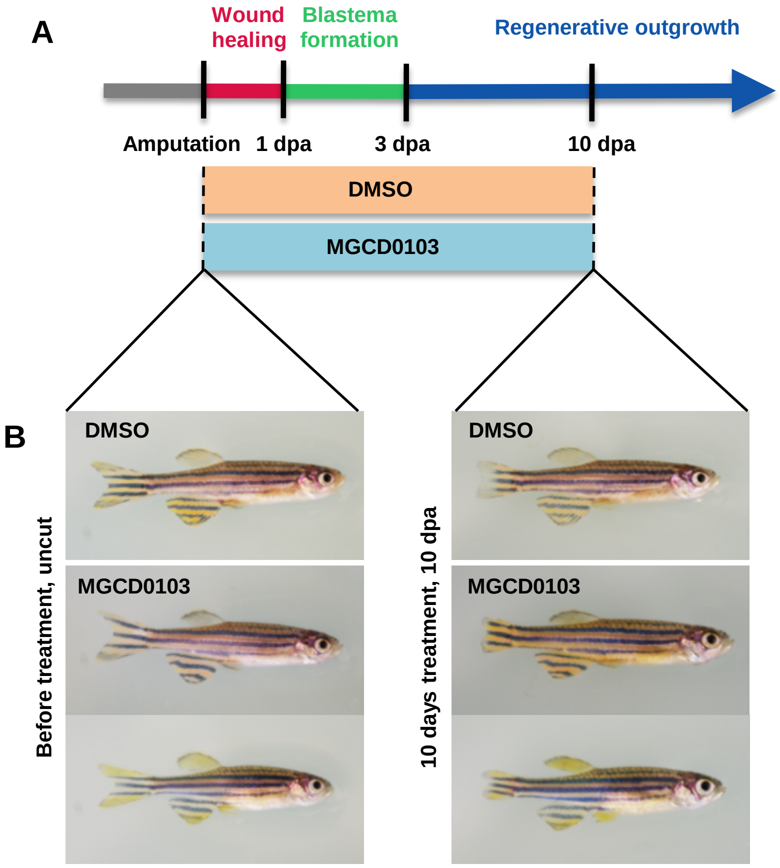

Figure S7. MGCD0103 treatment does not affect the general health of zebrafish.

(A) Schematic representation of fin regeneration progression and MGCD0103 treatment. Fish were treated with DMSO or MGCD0103 starting from the time of amputation. (B) Images of fish before and 10 days after DMSO or MGCD0103 treatment. The general health of fish treated with MGCD0103 for 10 days was not obviously altered compared with fish incubated with DMSO-containing water.

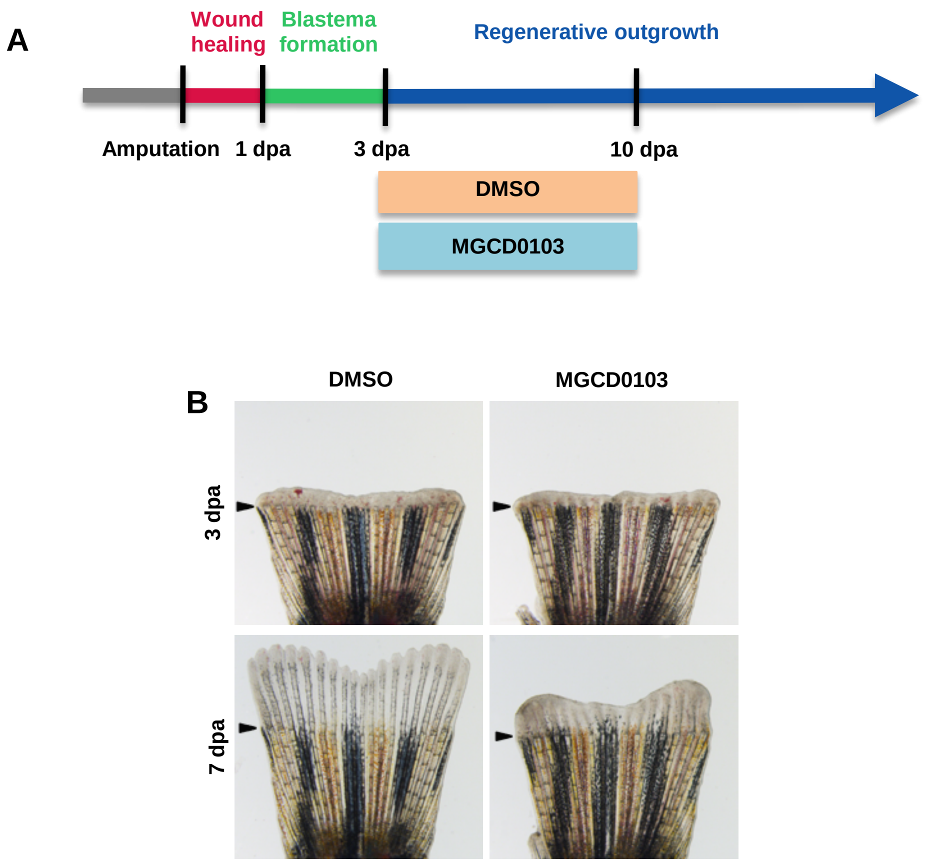

Figure S8. Hdac1 inhibition after blastema formation is sufficient to impair regenerative outgrowth.

(A) Schematic representation of fin regeneration progression and MGCD0103 treatment. Fish were treated with DMSO or MGCD0103 starting from 3 dpa. (B) Whole caudal fins at 3 dpa and at 7 dpa treated with DMSO or 5 μM MGCD0103 starting from 3 dpa. Arrowheads indicate the amputation plane. Scale bar: 100 μm.

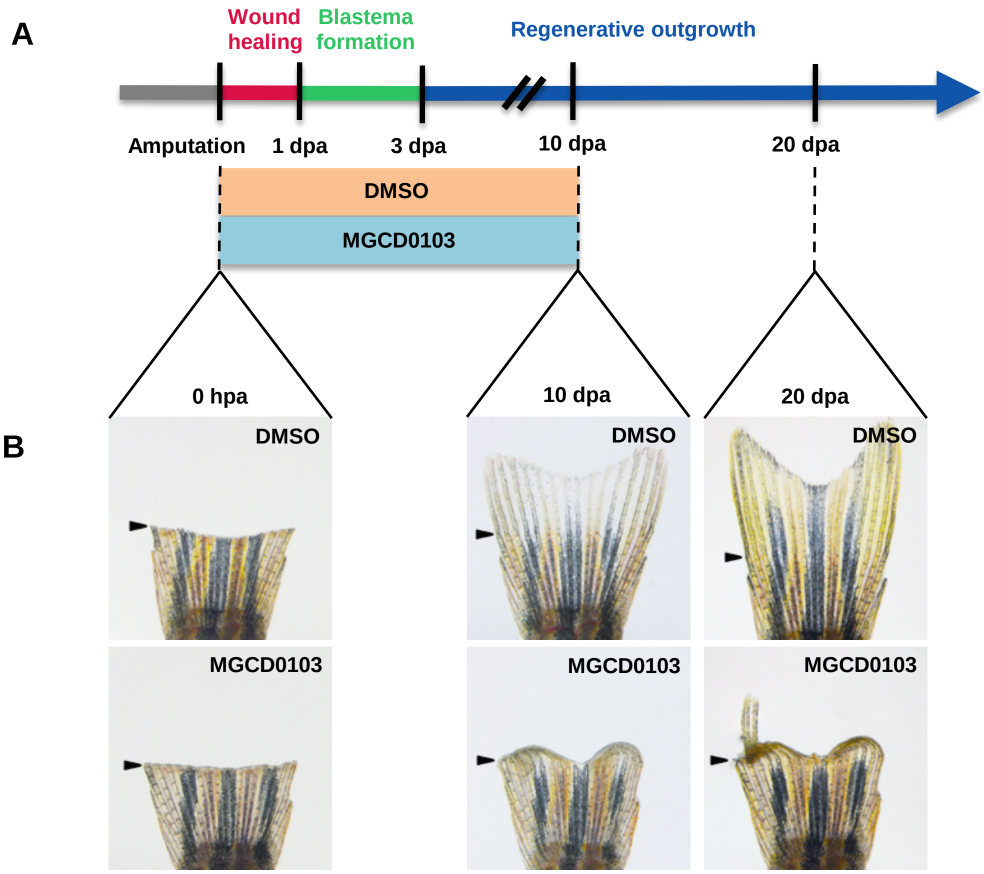

Figure S9. The effects of MGCD0103 treatment are not reversible.

(A) Schematic representation of fin regeneration progression and MGCD0103 treatment. Fish were treated with DMSO or MGCD0103 for 10 days starting from the time of amputation and then fish were transferred into normal fish water for 10 additional days. (B) Corresponding images of whole caudal fins just after amputation (0 hpa), at 10 dpa or at 20 dpa. Effects of MGCD0103 treatment were not reversible. In some cases, a few rays were able to resume the regeneration process. Arrowheads indicate the amputation plane.

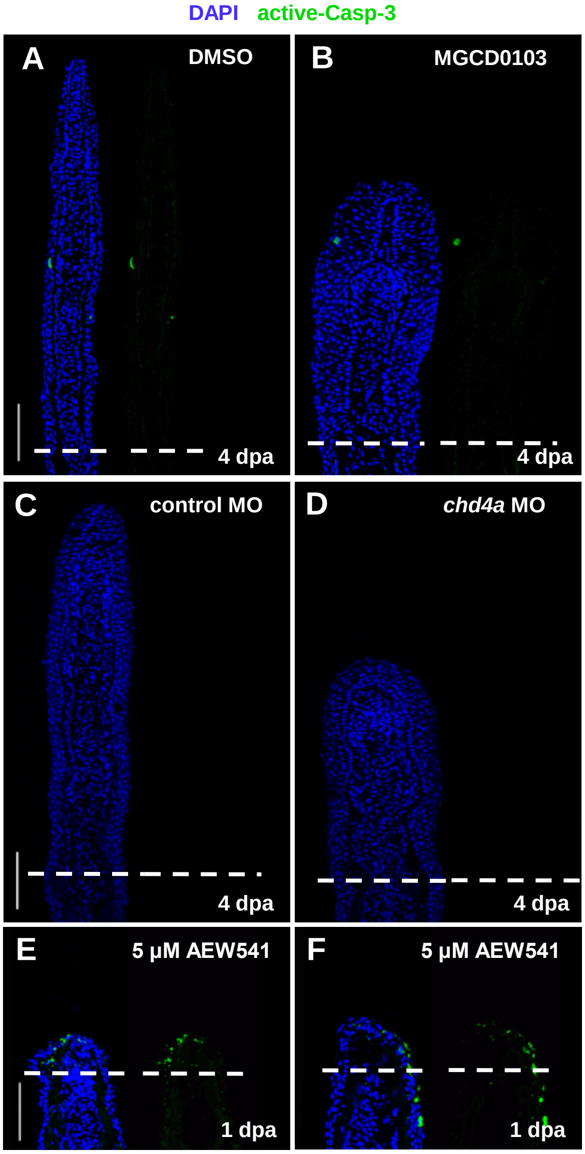

Figure S10. Hdac1 inhibition and *chd4a* knockdown do not result in the activation of the apoptosis marker caspase-3.

(A-F) Longitudinal sections of fin regenerates stained with active caspase-3 antibody (green) and with DAPI (blue). Only a few cells positive for active caspase-3 are present in control fins (A,C). Fins treated with MGCD0103 (B) or injected with *chd4a* MO (D) do not display any increase of apoptosis at 4 dpa. (E-F) As a positive control, the activation of caspase-3 was assessed in fin regenerates at 1 dpa treated with the chemical inhibitor of Igf1 receptor NVP_AEW541. Inhibition of IGF signaling blocks fin regeneration and results in the increase of cell apoptosis in the wound epidermis (Chablais et al., 2010). Dashed lines indicate the amputation plane. Scale bars: 100 µm.

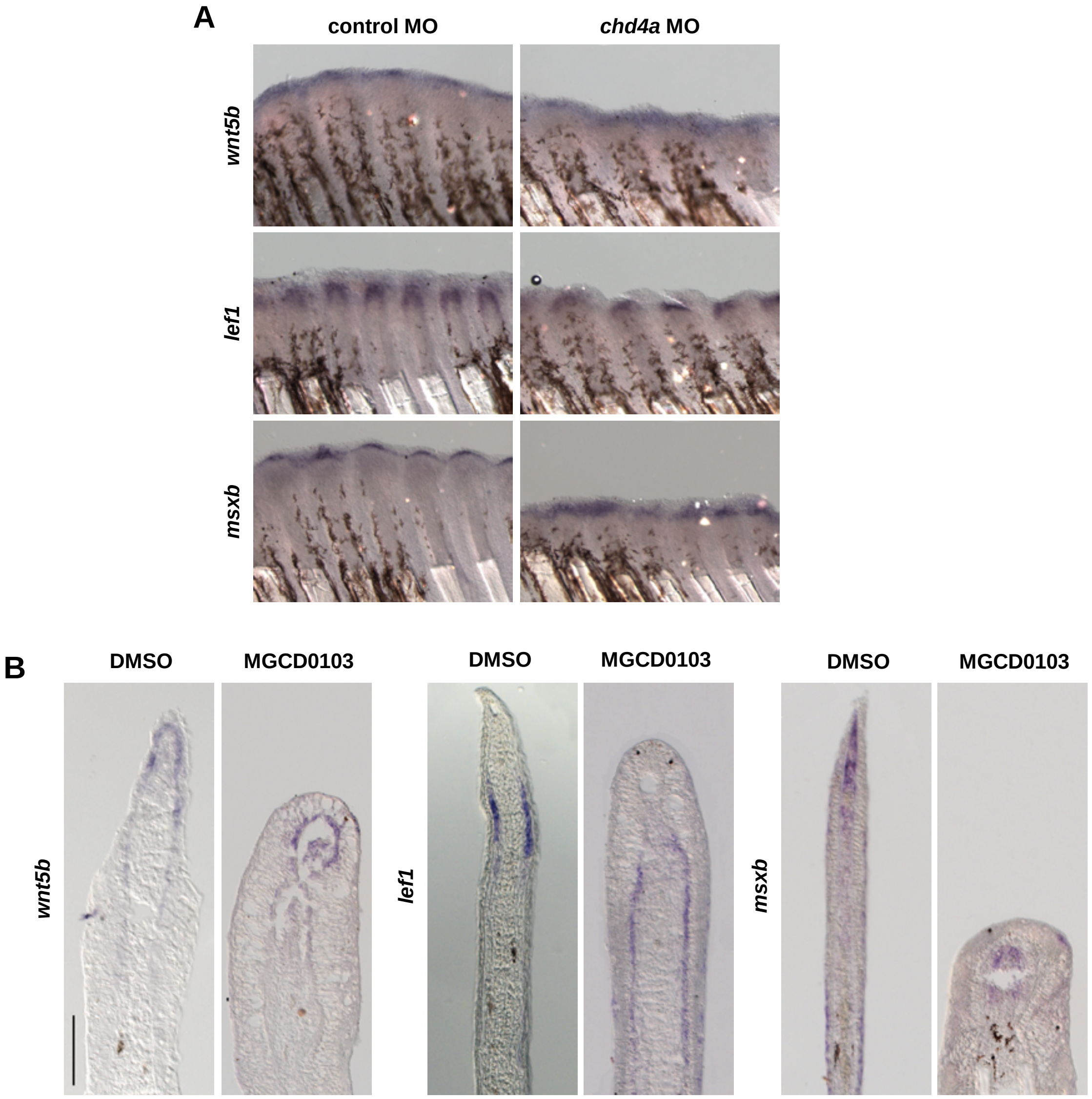

Figure S11. *wnt5b*, *lef1* and *msxb* are expressed in *chd4a* MO-injected and MGCD0103-treated fins.

(A-B) *In situ* hybridization with *wnt5b*, *lef1* and *msxb* mRNA antisense probes in *chd4a* MO-injected (A) and in MGCD0103-treated (B) fin regenerates at 4 dpa. Scale bar: 100 µm.

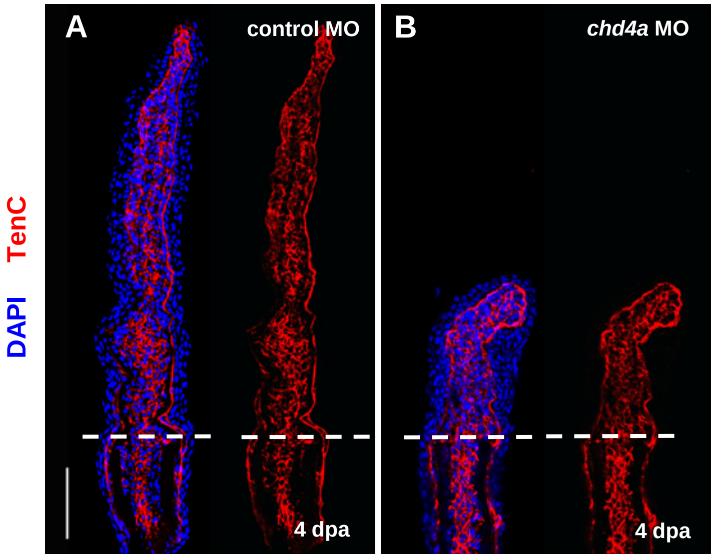

Figure S12. ***chd4a*** **knockdown does not affect Tenascin C expression.**

(A-B) Longitudinal sections of fin regenerates at 4 dpa injected with control (A) or *chd4a* (B) MOs stained with Tenascin C antibody (red) and with DAPI (blue). Mesenchymal remodeling was not altered in *chd4a* MO-injected fins. Dashed lines indicate the amputation plane. Scale bars: 100 µm.

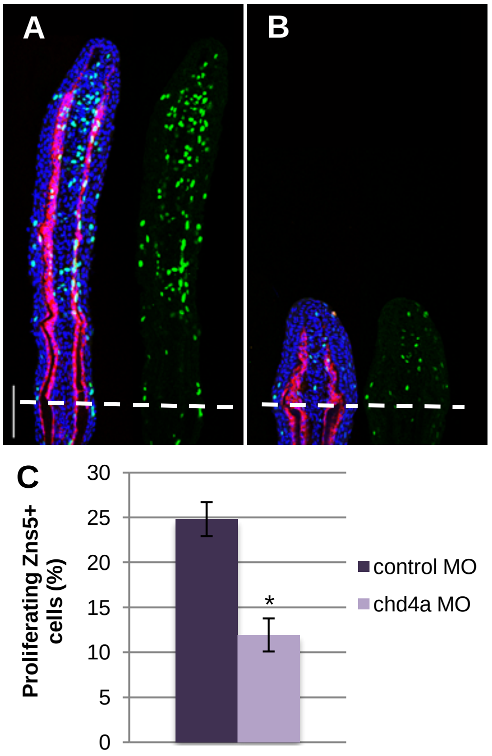

Figure S13. *chd4a* knockdown reduces osteoblast proliferation.

(A-B) Longitudinal sections of fin regenerates at 4 dpa injected with control (A) or *chd4a* (B) MOs triply stained with BrdU (green), ZNS5 antibody (red) and DAPI (blue). Dashed lines indicate the amputation plane. Scale bar: 100 μm. (C) Percentage of ZNS5-positive cells that were also positive for BrdU relative to the total number of ZNS5-positive cells in fin regenerates at 4 dpa injected with with control or *chd4a* MOs. Error bars represent the s.e.m. n = 17. * P<0.01.

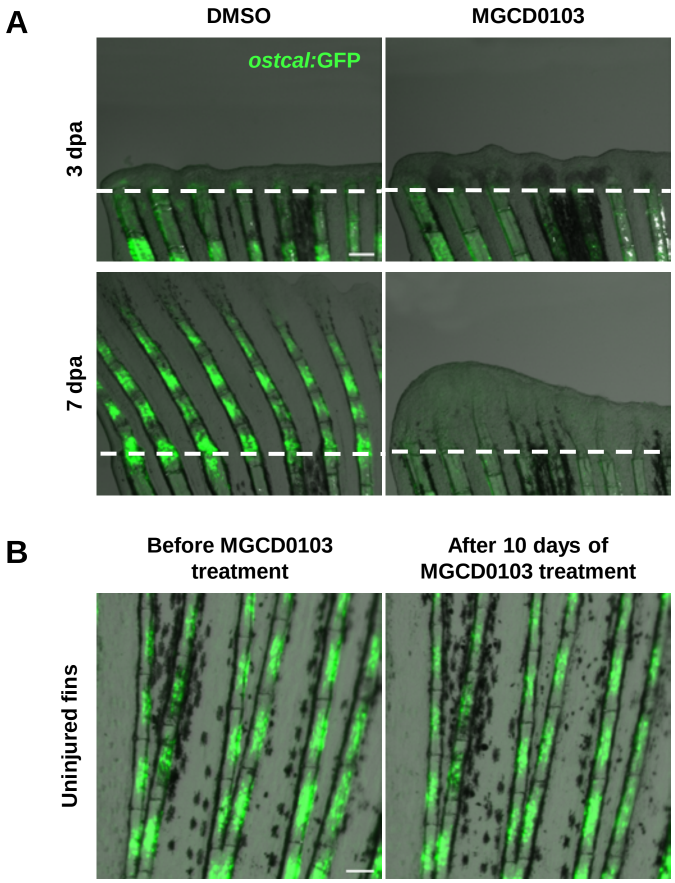

Figure S14. Hdac1 inhibition after blastema formation is sufficient to block reactivation of *osteocalcin* expression.

(A) Caudal fins of *osteocalcin:*GFP transgenic fish at 3 dpa and 7 dpa treated with DMSO or MGCD0103 starting at 3 dpa. *osteocalcin:*GFP expression was not reactivated in fin regenerates treated with MGCD0103 after blastema has been formed. (B) Uninjured caudal fins of *osteocalcin:*GFP transgenic fish before and 10 days after MGCD0103 treatment. MGCD0103 treatment does not alter *osteocalcin:*GFP expression in uninjured fins. Constant exposure times were used. Scale bars: 100 μm.
